# Supplementary material for: Microwave-Assisted Ionic Liquid-Catalyzed Selective Monoesterification of Alkylphosphonic Acids—An Experimental and a Theoretical Study
Source: Molecules. 2021 Aug 31;26(17):5303. doi: 10.3390/molecules26175303 (PMC8434145; doi:10.3390/molecules26175303)
Supplement: Supplementary file 1 [file molecules-26-05303-s001.zip › molecules-1347178-supplementary.pdf]

Supporting Information

Microwave-Assisted Ionic Liquid-Catalyzed Selective Monoesterification of Alkylphosphonic Acids – an Experimental and a Theoretical Study

Nikoletta Harsági, Réka Henyecz, Péter Ábrányi-Balogh, László Drahos and György Keglevich*

Content

1. ^{31}P, ^{13}C and ^{1}H NMR spectra for the new esters acids 3

*Monopropyl ethylphosphonate (**2Bb**)* 3

*Monobutyl ethylphosphonate (**2Bd**)* 4

*Monopropyl propylphosphonate (**2Cb**)* 6

*Monoethyl butylphosphonate (**2Da**)* 7

*Monopropyl butylphosphonate (**2Db**)* 9

*Monoisopropyl butylphosphonate (**2Dc**)* 10

*Monobutyl butylphosphonate (**2Dd**)* 12

2. Table S1–S5 containing the computed row data 14

3. XYZ geometries of computed species 18

1. ^{31}P, ^{13}C and ^{1}H NMR spectra for the new esters acids

*Monopropyl ethylphosphonate (**2Bb**)*

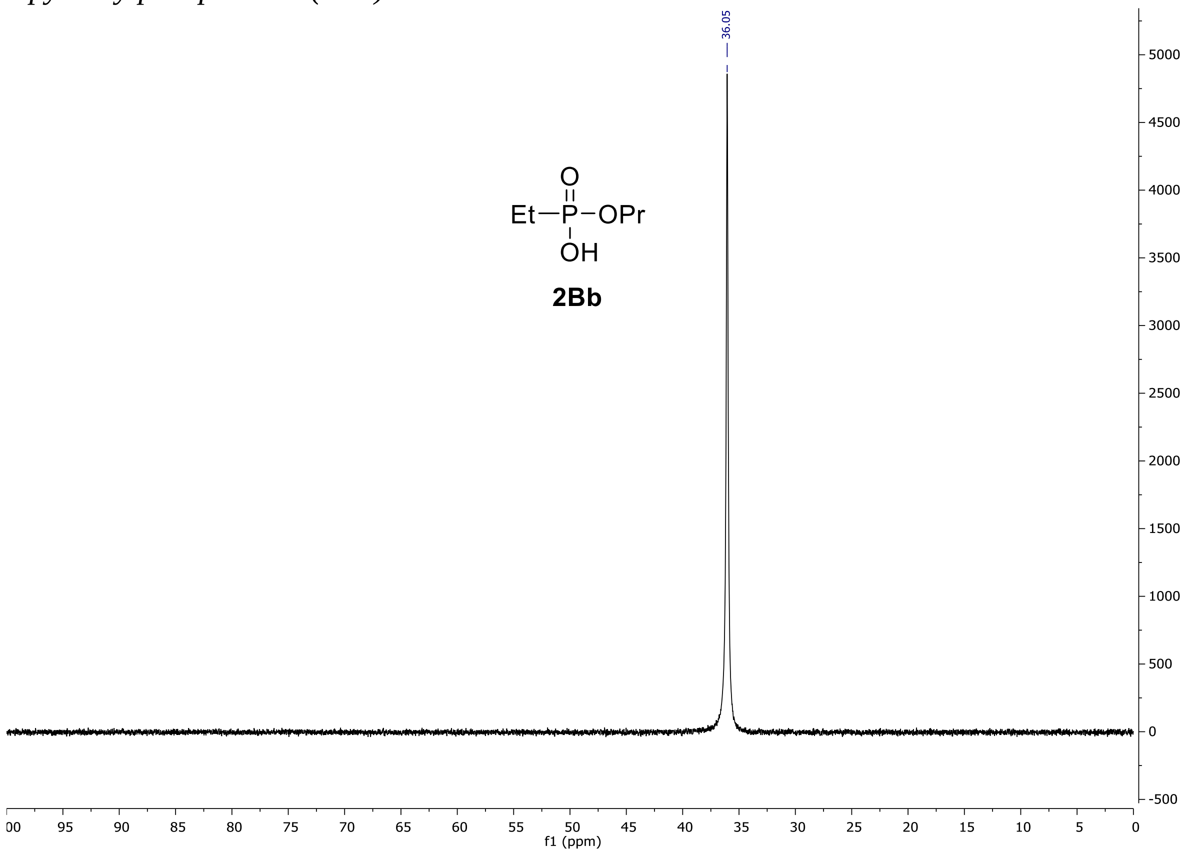

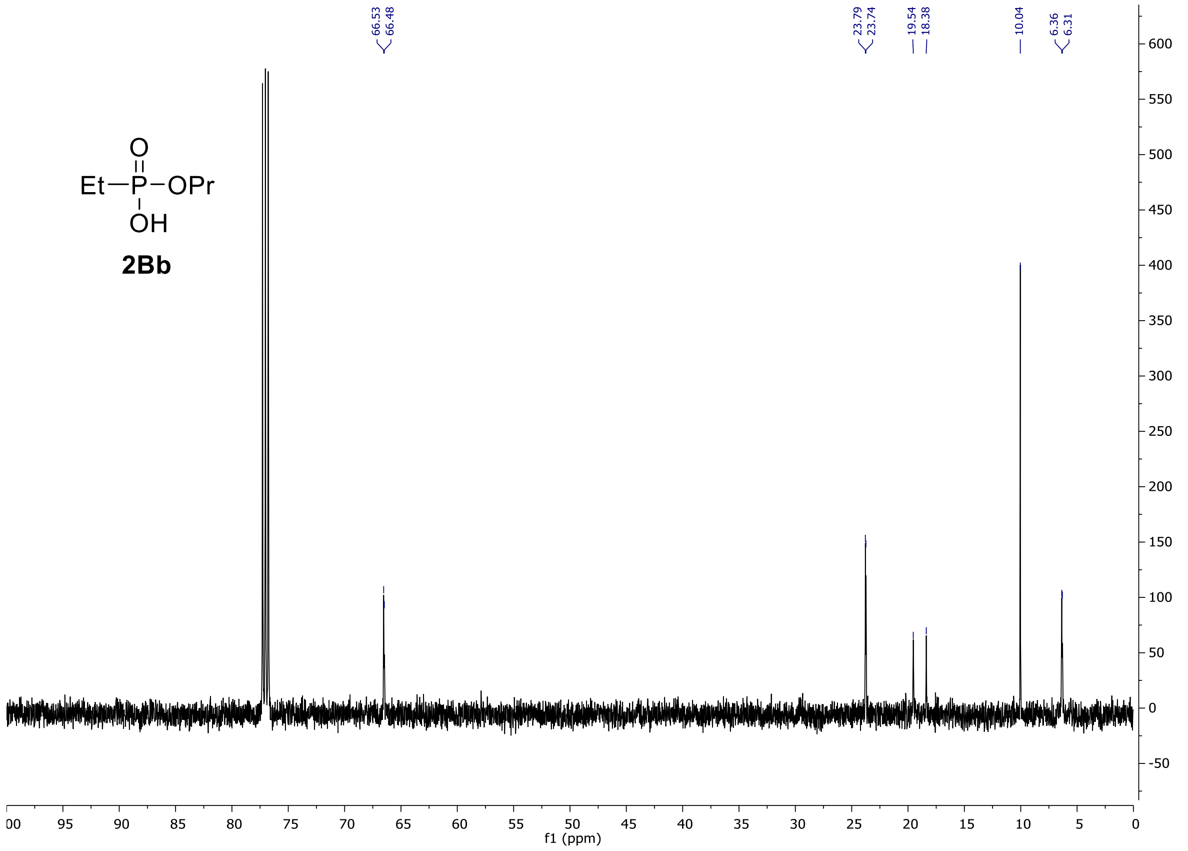

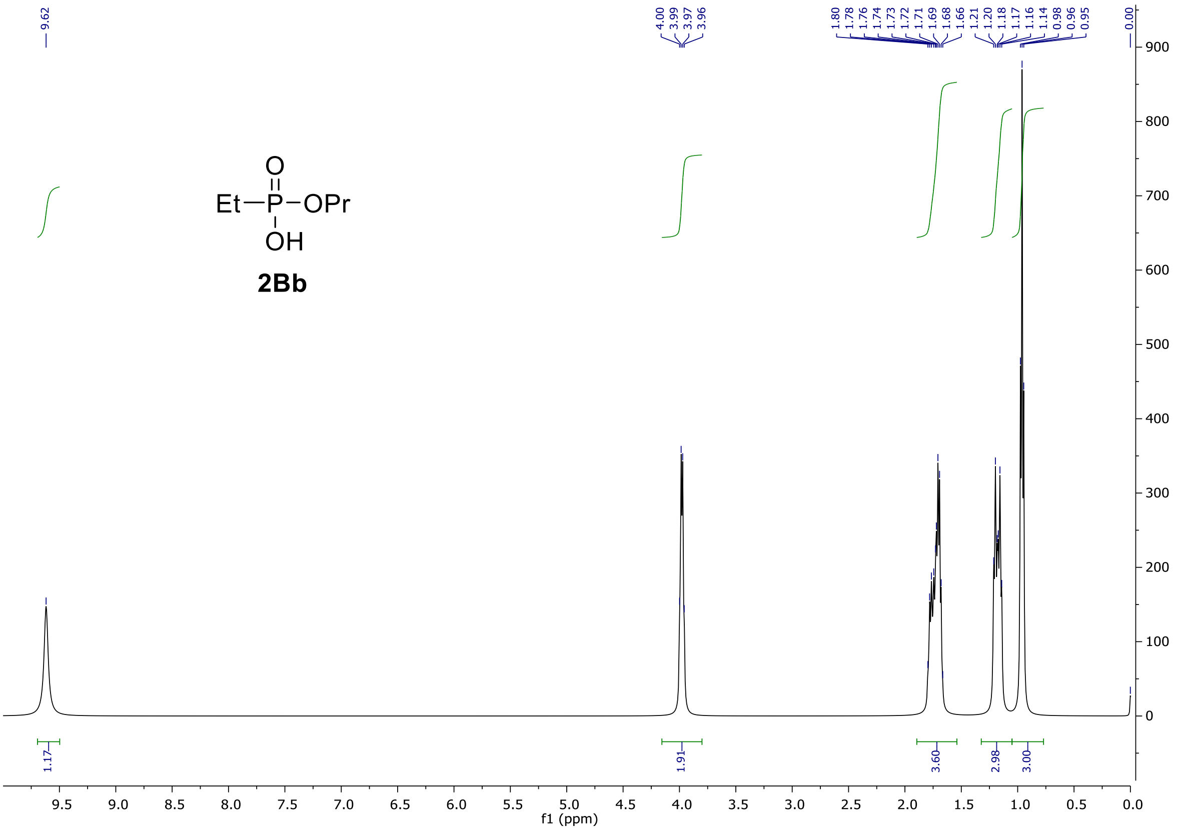

*Monobutyl ethylphosphonate (**2Bd**)*

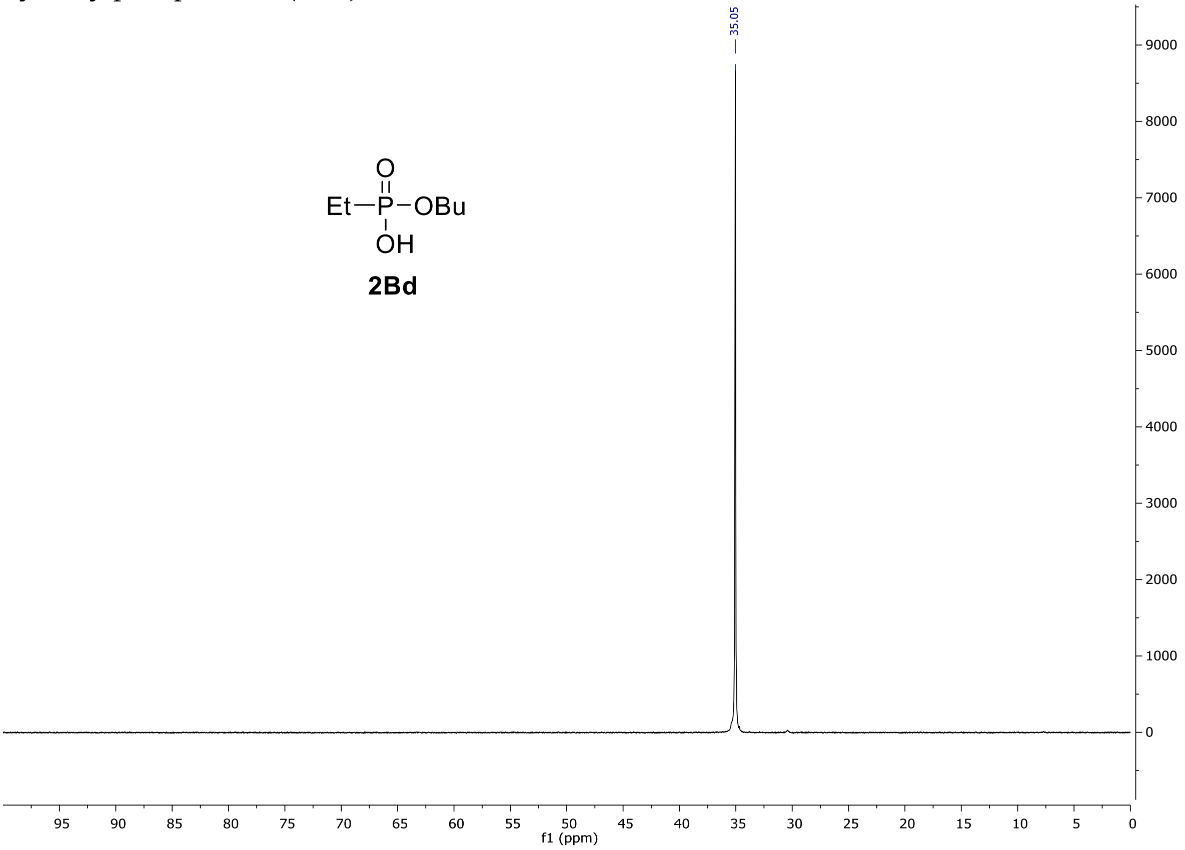

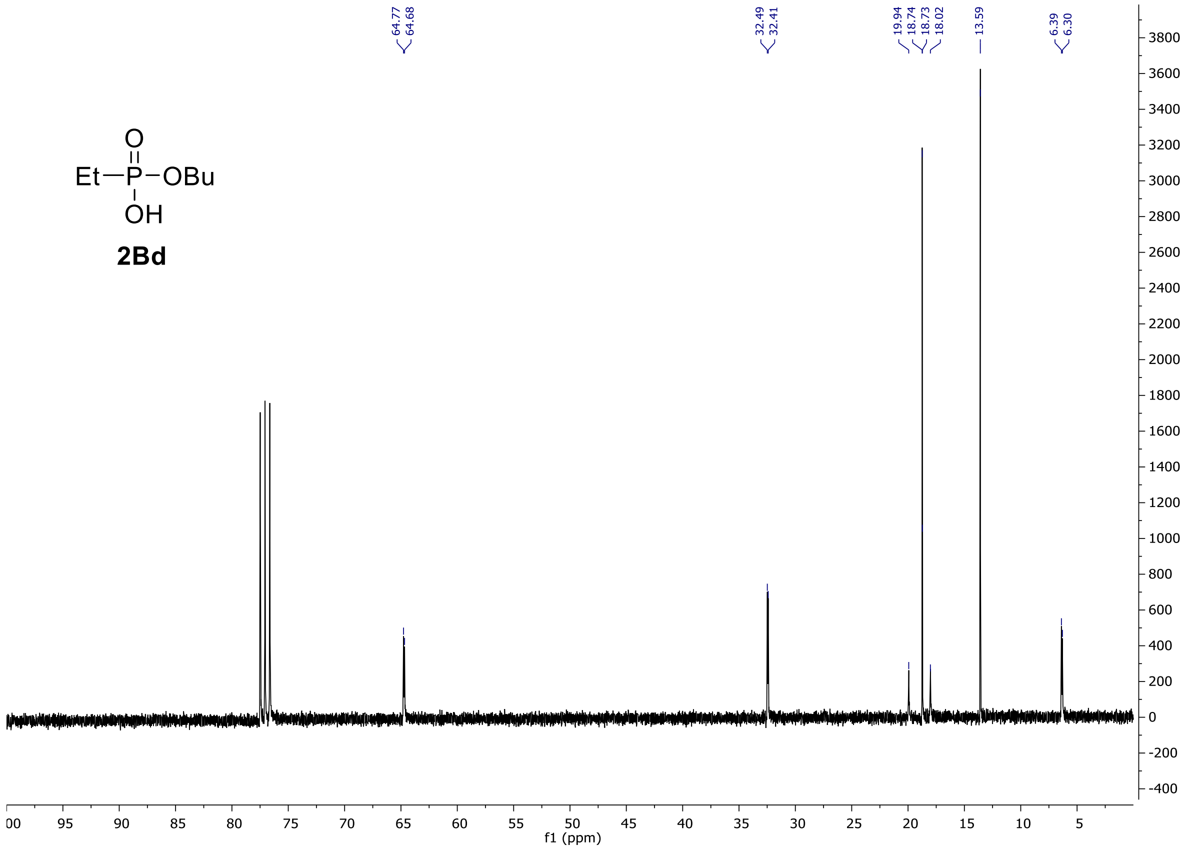
Et—P(=O)(OH)—OBu
2Bd
64.77
64.68
32.49
32.41
19.94
18.74
18.73
18.02
13.59
6.39
6.30
f1 (ppm)

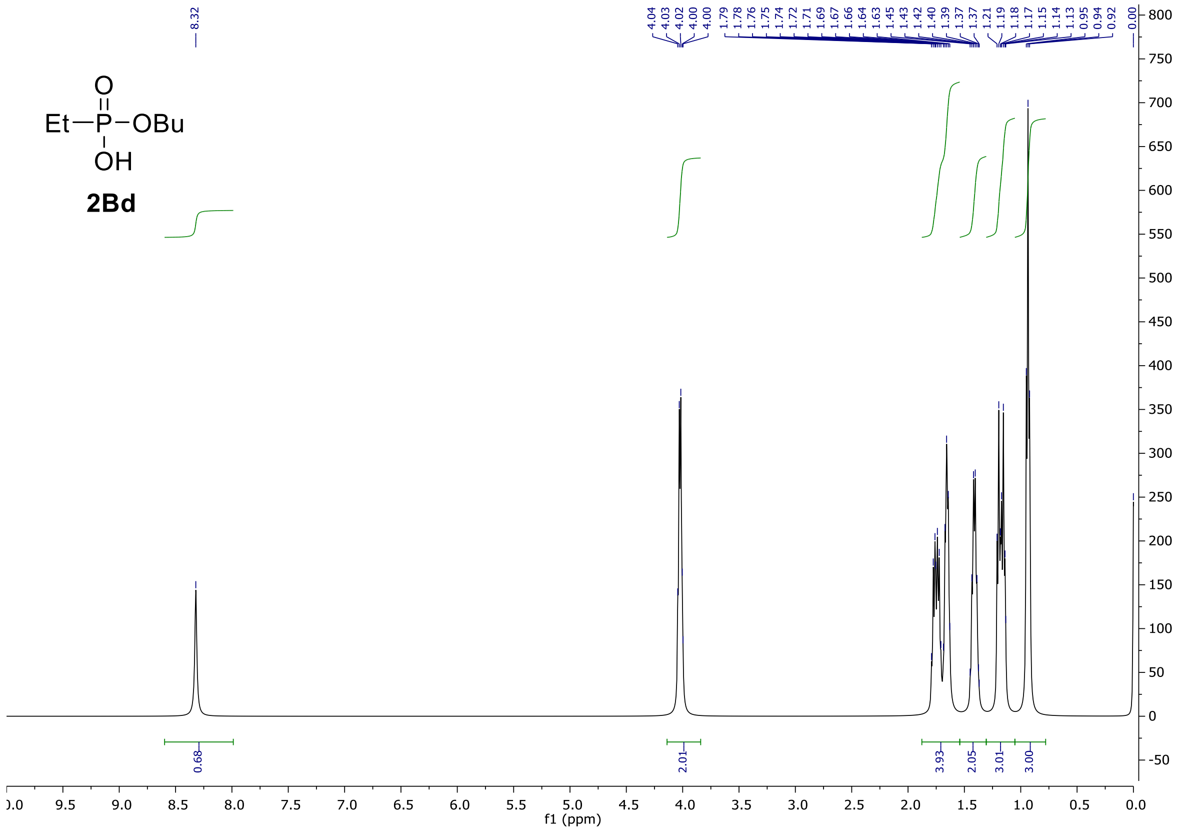
Et—P(=O)(OH)—OBu
2Bd
8.32
4.04
4.03
4.02
4.00
4.00
4.00
1.79
1.78
1.76
1.75
1.74
1.72
1.71
1.69
1.67
1.66
1.64
1.63
1.45
1.43
1.42
1.40
1.39
1.37
1.37
1.21
1.19
1.18
1.17
1.15
1.14
1.13
0.95
0.94
0.92
0.00
0.68
2.01
3.93
2.05
3.01
3.00
f1 (ppm)

*Monopropyl propylphosphonate (**2Cb**)*

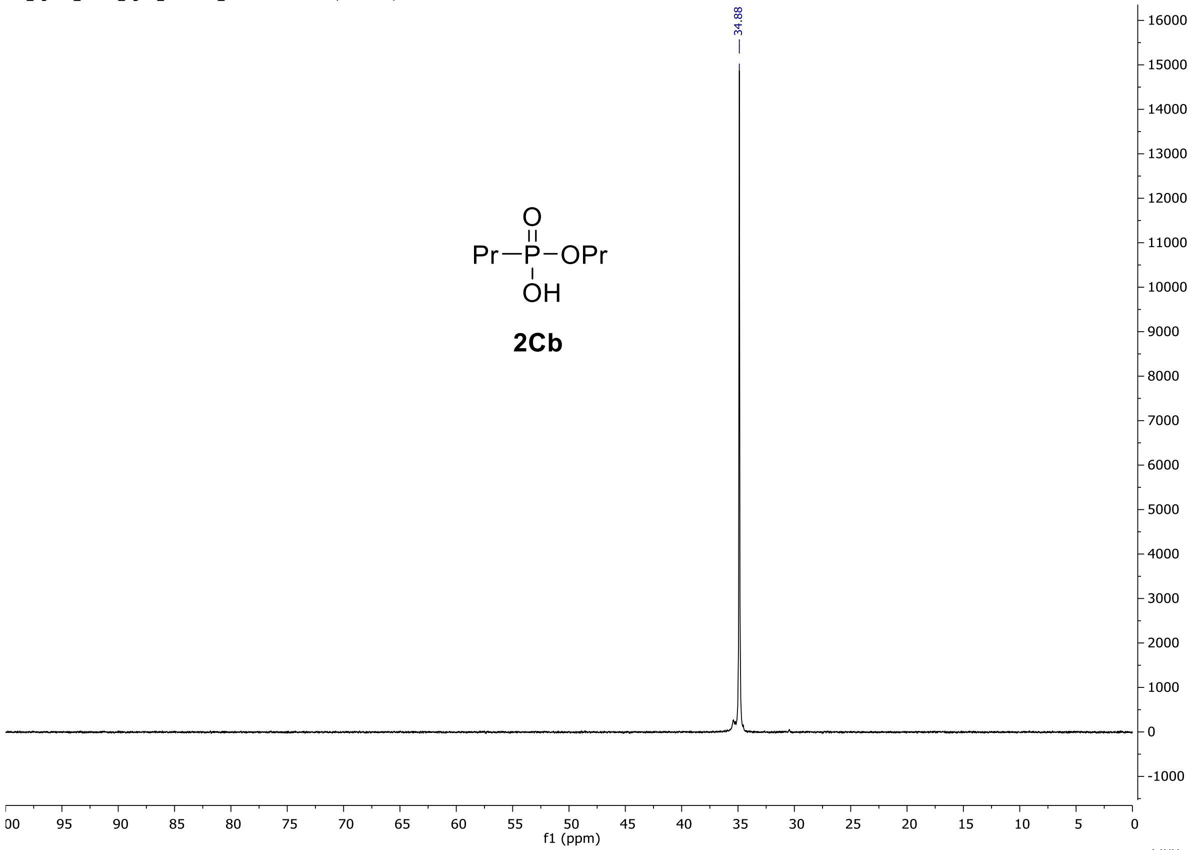

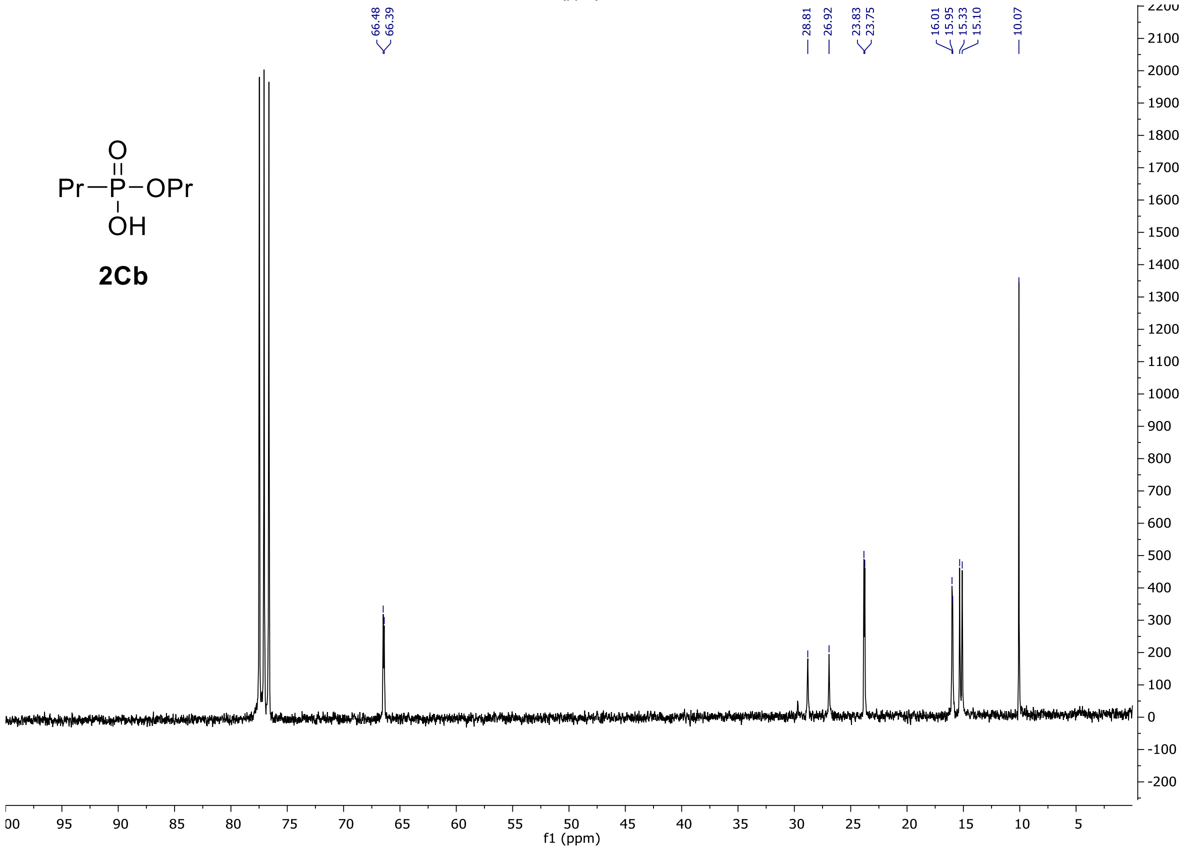

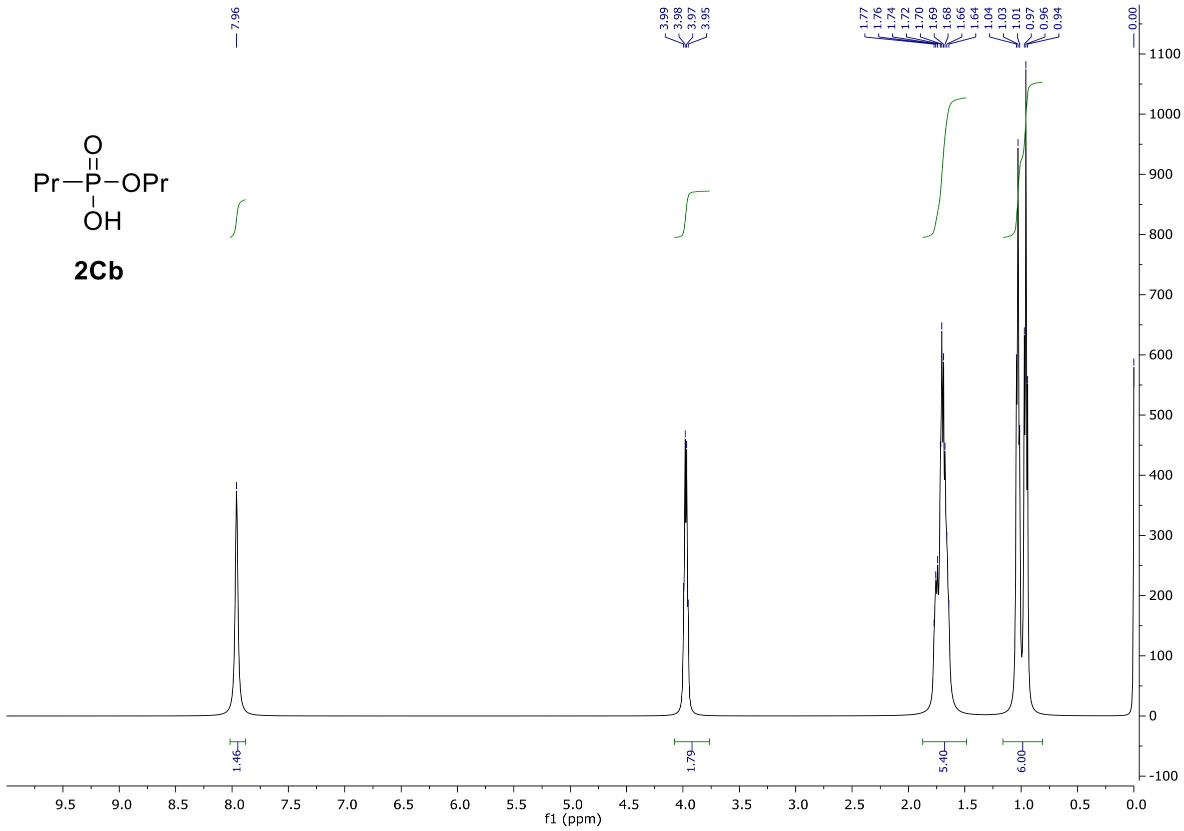

*Monoethyl butylphosphonate (**2Da**)*

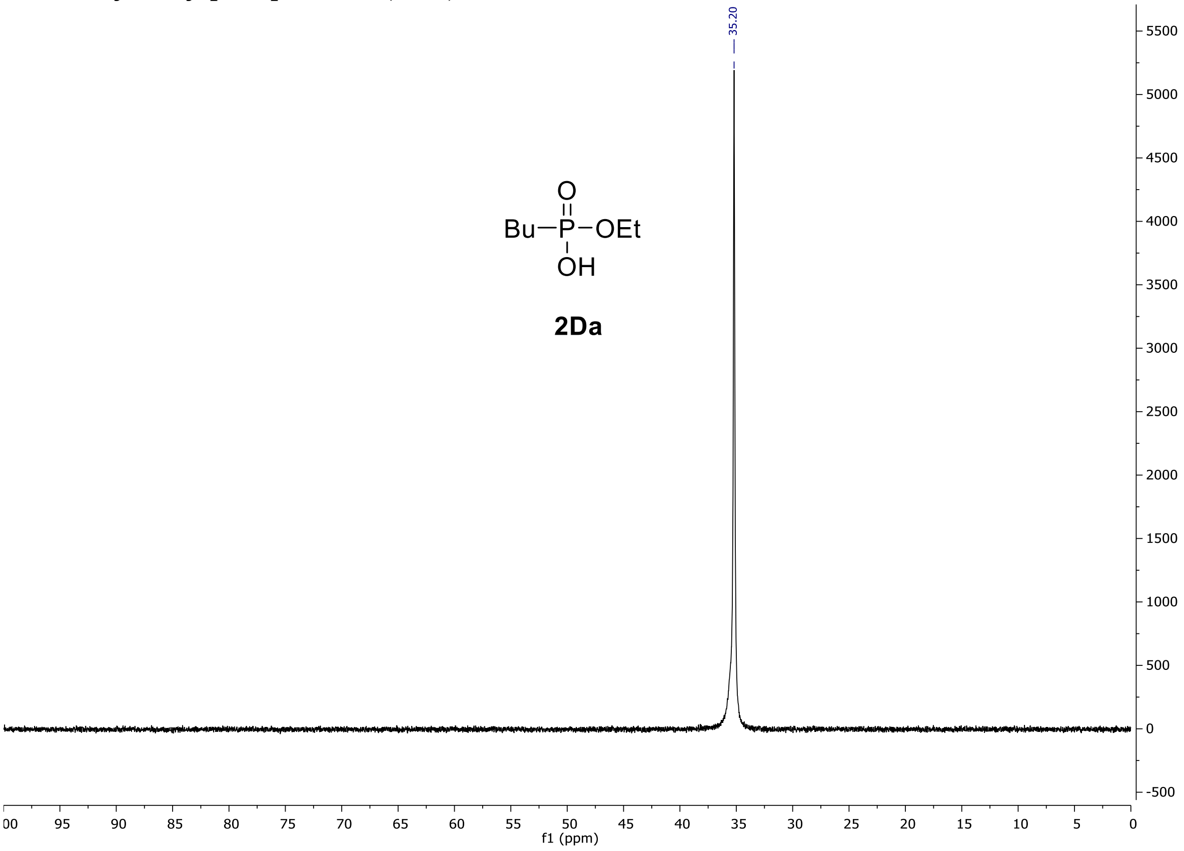

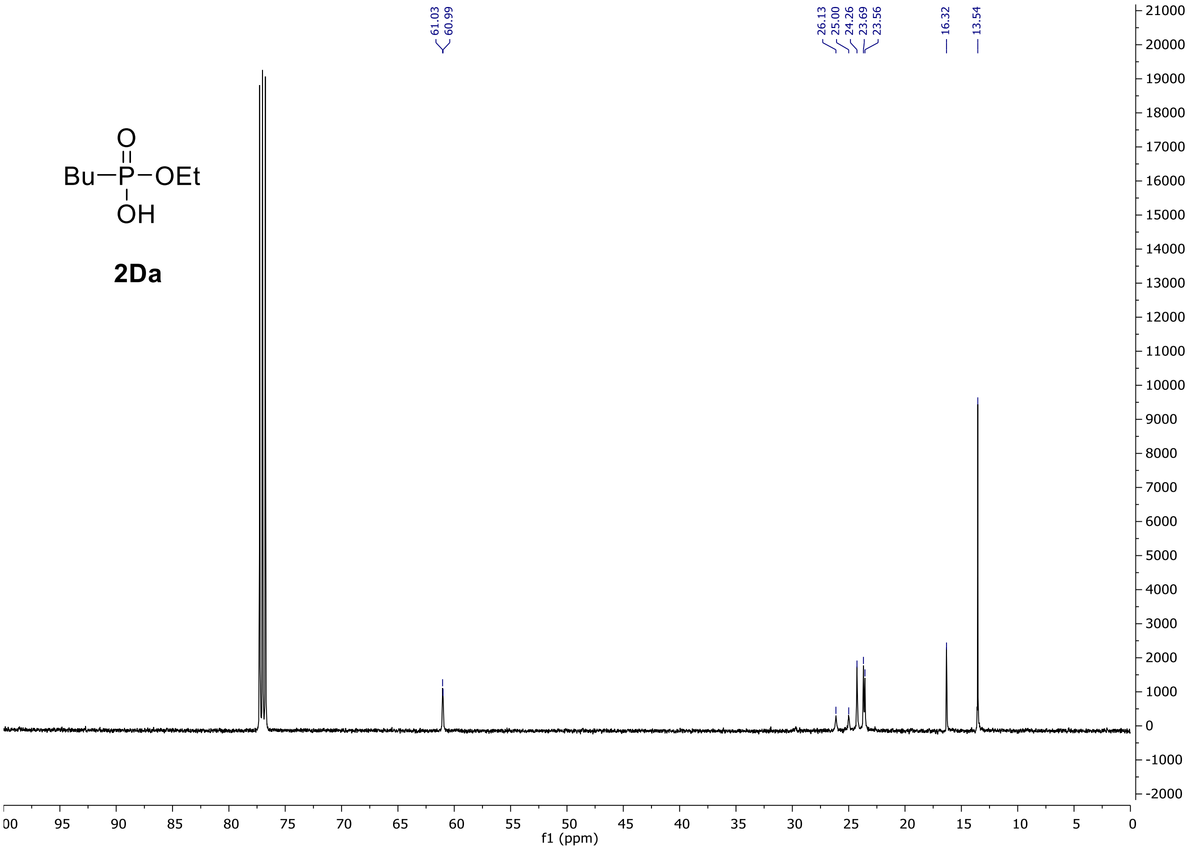
Bu–P(=O)(OH)–OEt
2Da
61.03
60.99
26.13
25.00
24.26
23.69
23.56
16.32
13.54
f1 (ppm)

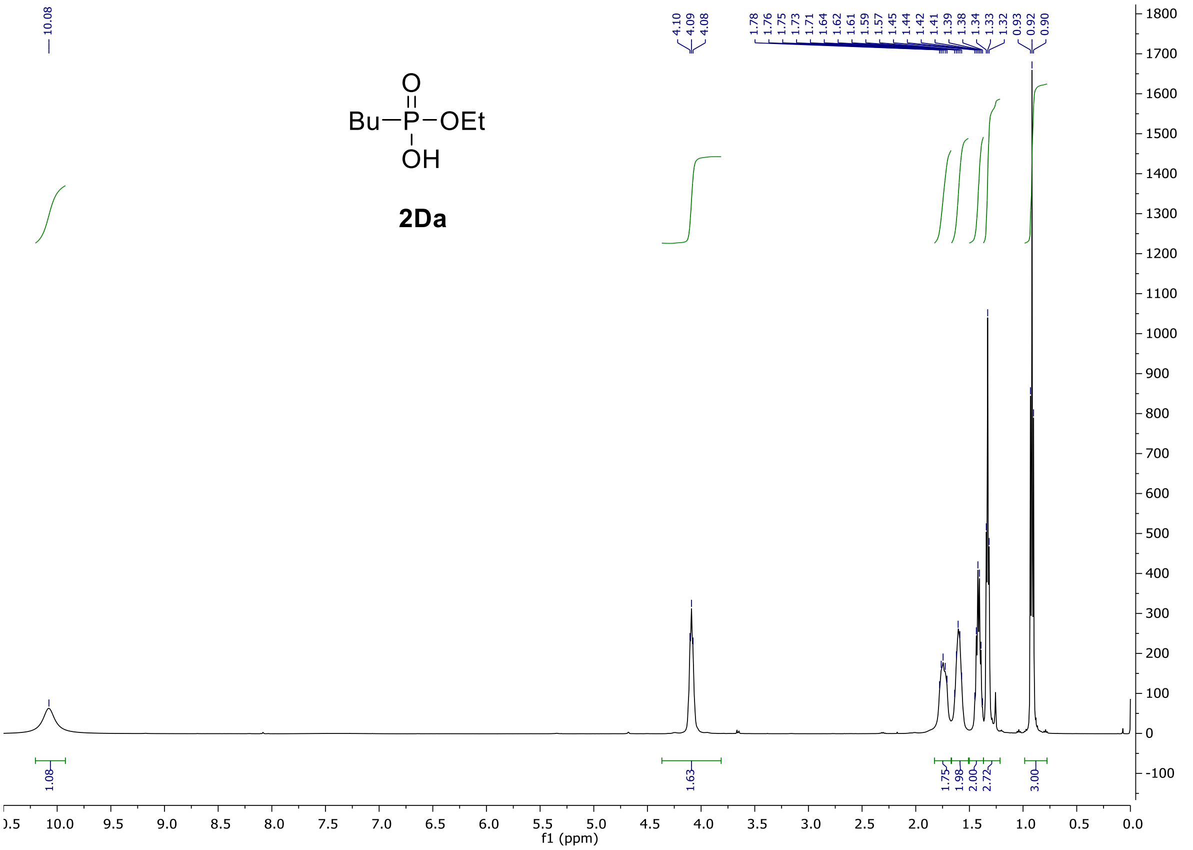
Bu–P(=O)(OH)–OEt
2Da
10.08
4.10
4.09
4.08
1.78
1.76
1.75
1.73
1.71
1.64
1.62
1.61
1.59
1.57
1.45
1.44
1.42
1.41
1.39
1.38
1.34
1.33
1.32
0.93
0.92
0.90
1.08
1.63
1.75
1.98
2.00
2.72
3.00
f1 (ppm)

*Monopropyl butylphosphonate (**2Db**)*

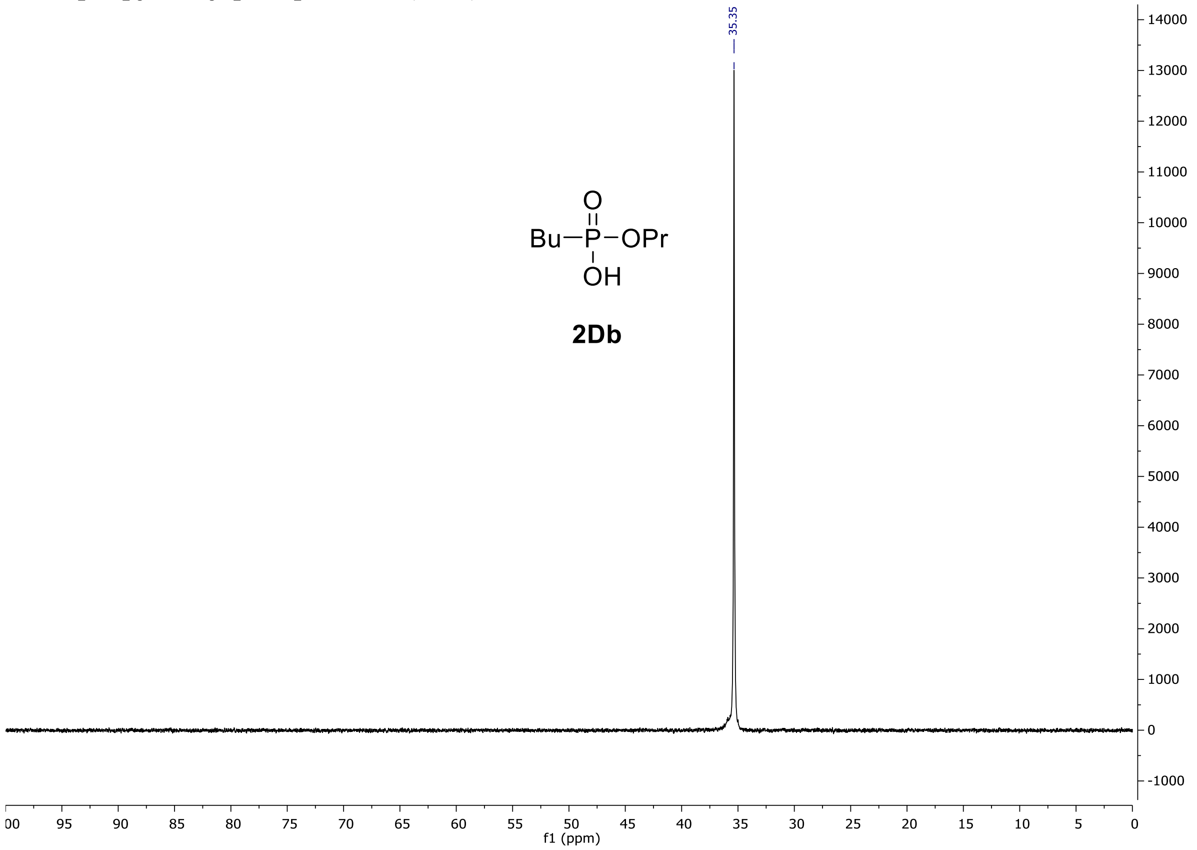

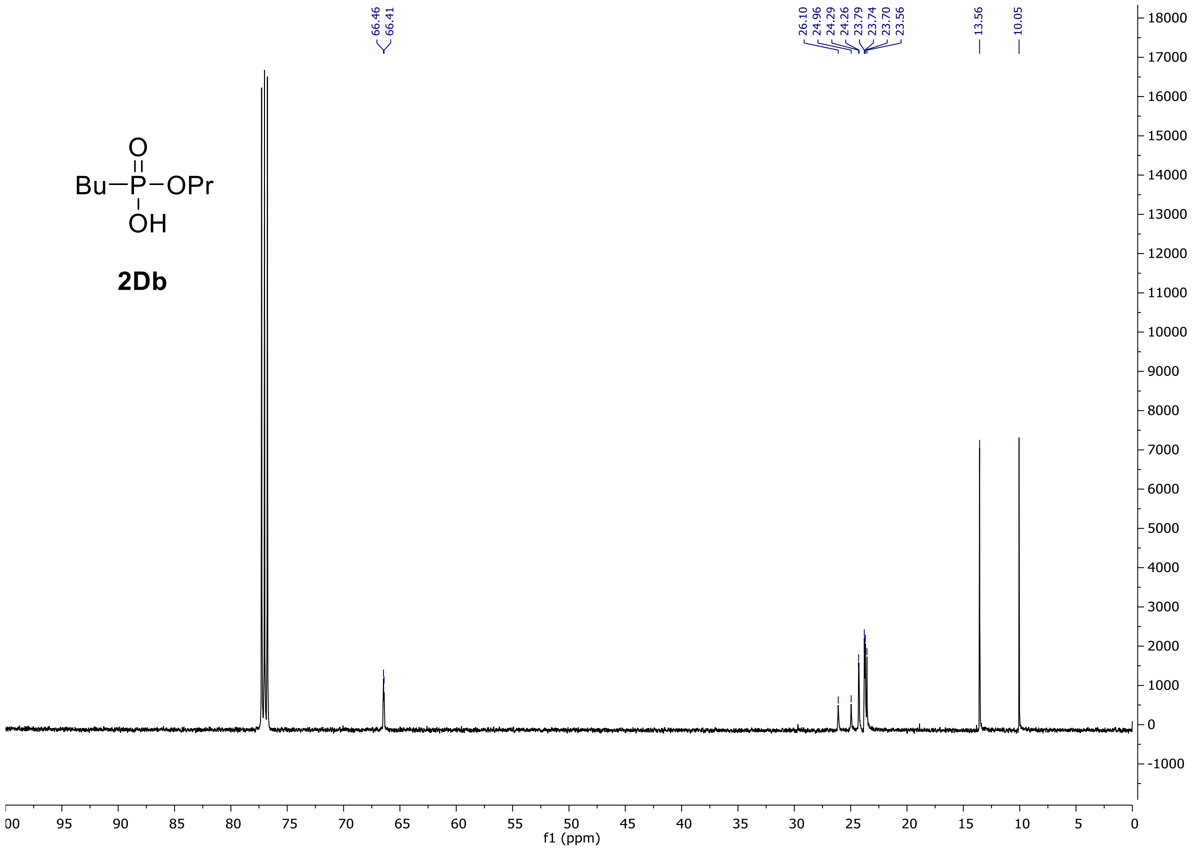

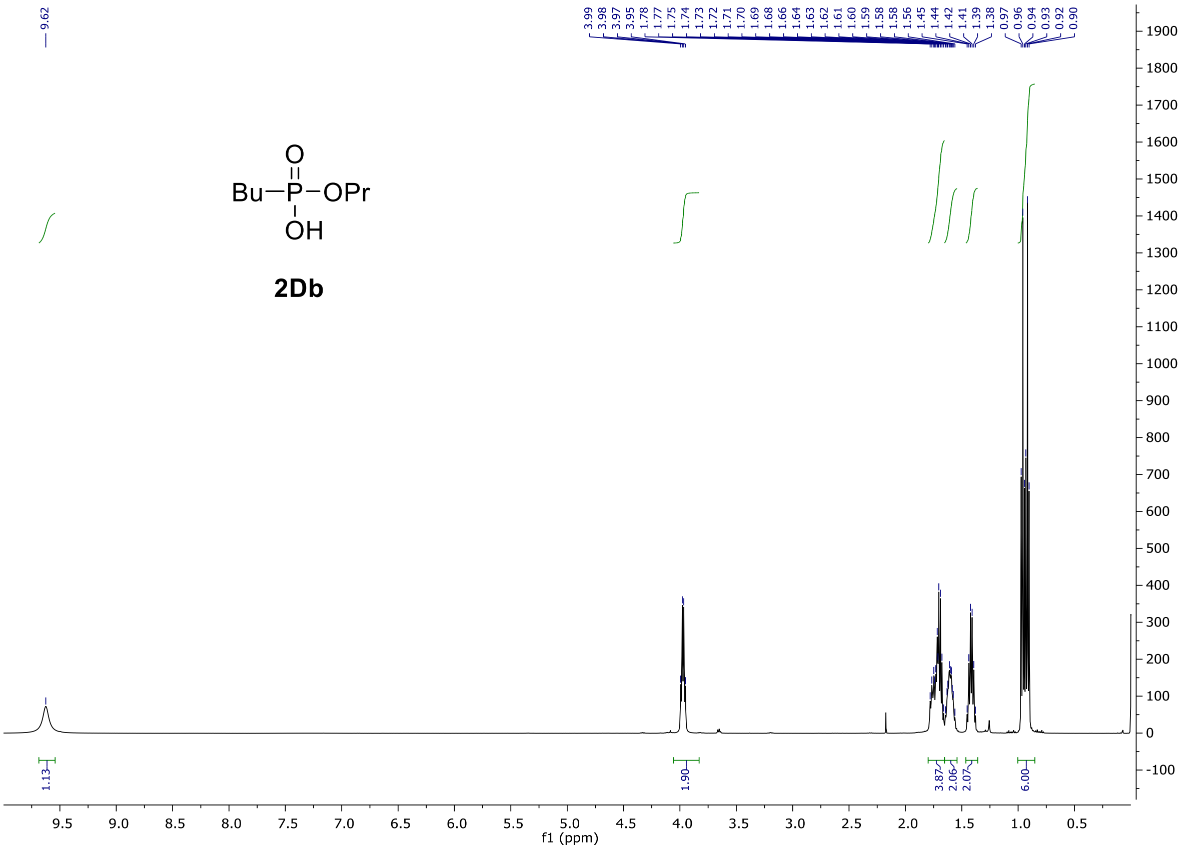

*Monoisopropyl butylphosphonate (**2Dc**)*

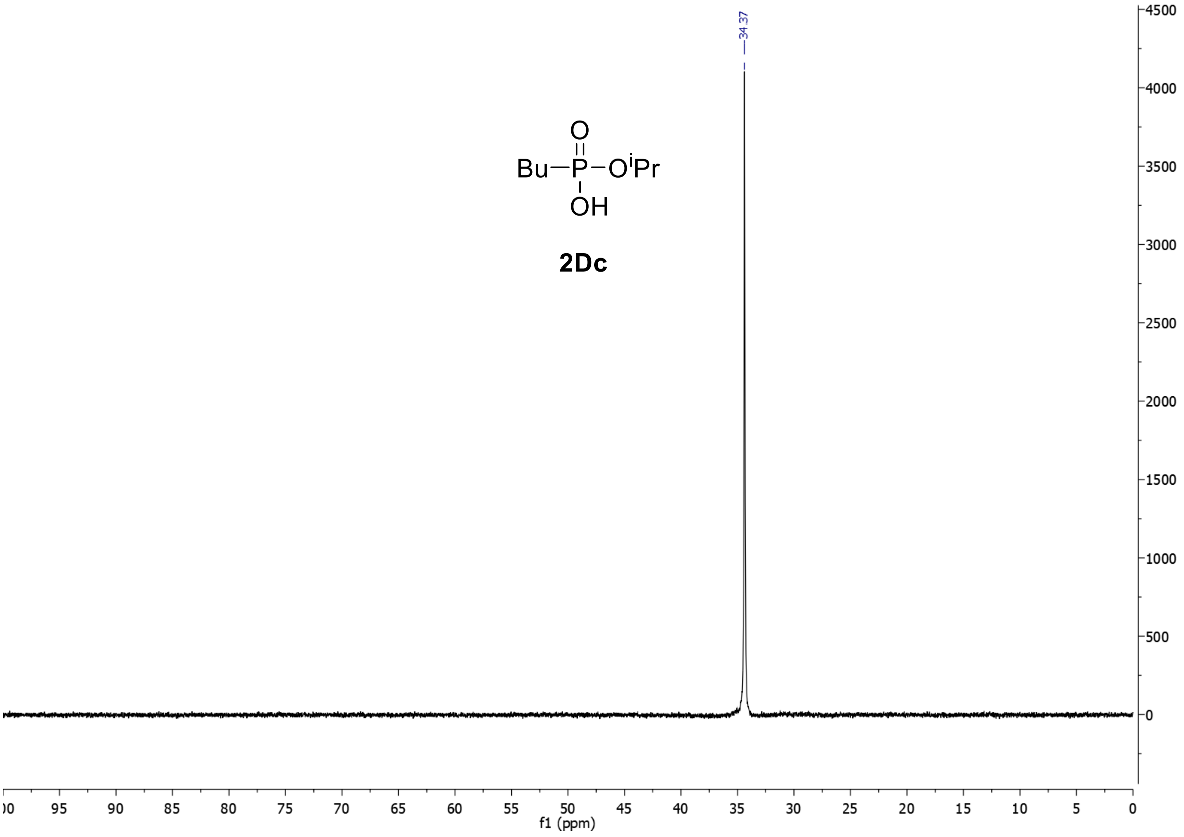

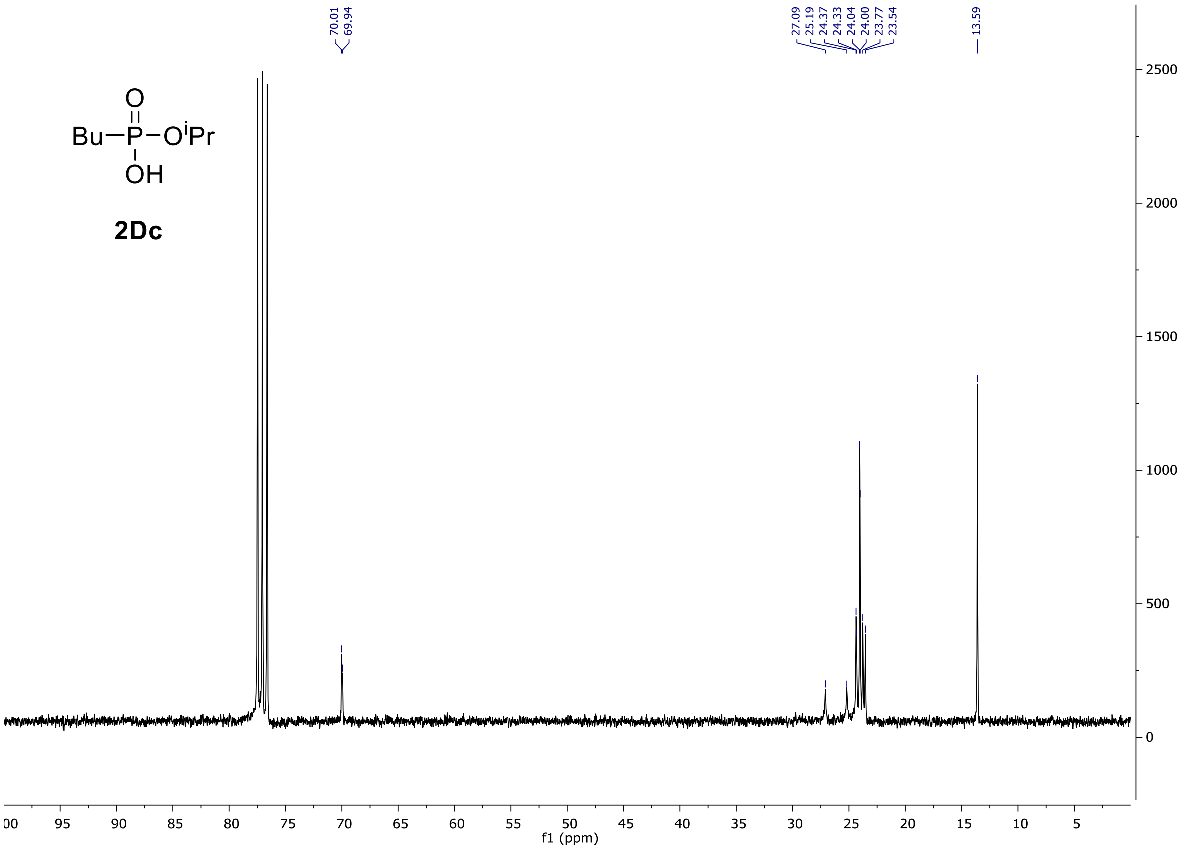
Bu−P(=O)(OH)−O^{i}Pr
2Dc
70.01
69.94
27.09
25.19
24.37
24.33
24.04
24.00
23.77
23.54
13.59
f1 (ppm)

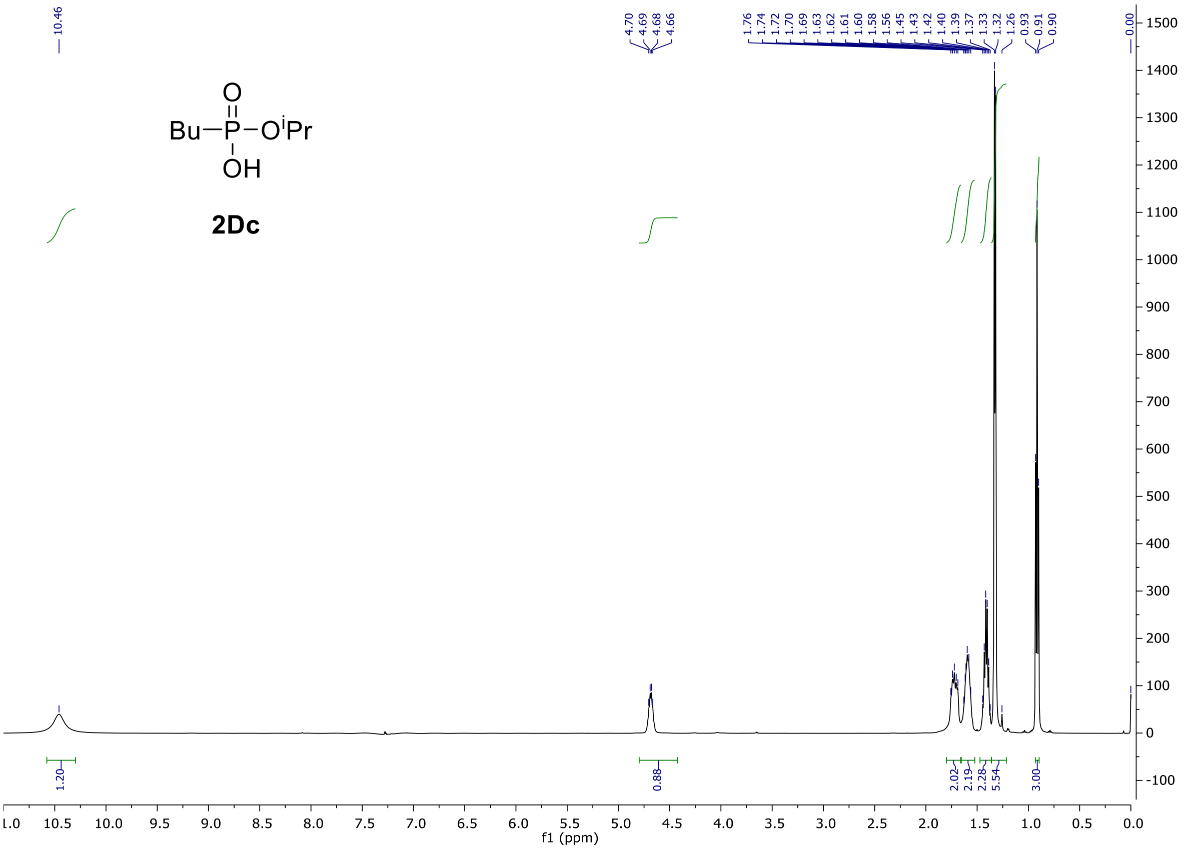
Bu−P(=O)(OH)−O^{i}Pr
2Dc
10.46
4.70
4.69
4.68
4.66
1.76
1.74
1.72
1.70
1.69
1.63
1.62
1.61
1.60
1.58
1.56
1.45
1.43
1.42
1.40
1.39
1.37
1.33
1.32
1.26
0.93
0.91
0.90
0.00
1.20
0.88
2.02
2.19
2.28
5.54
3.00
f1 (ppm)

*Monobutyl butylphosphonate (**2Dd**)*

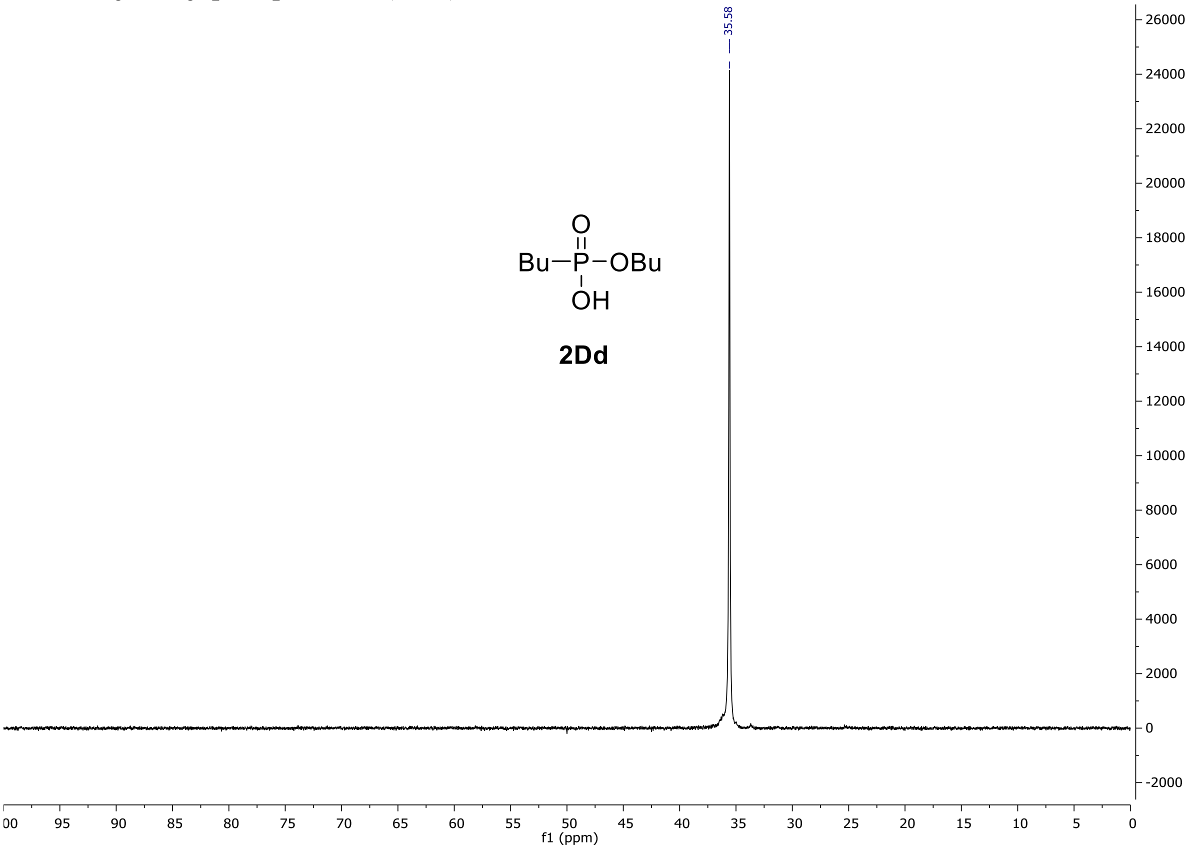

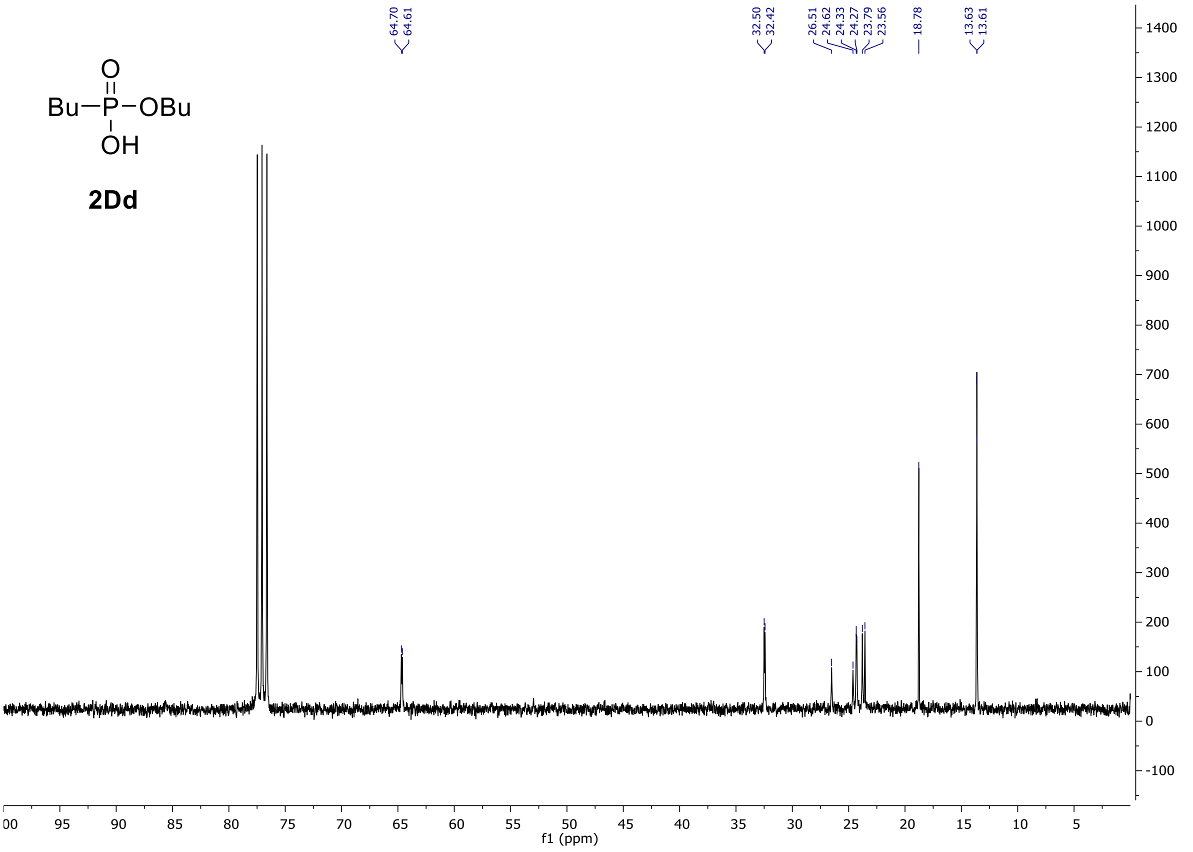

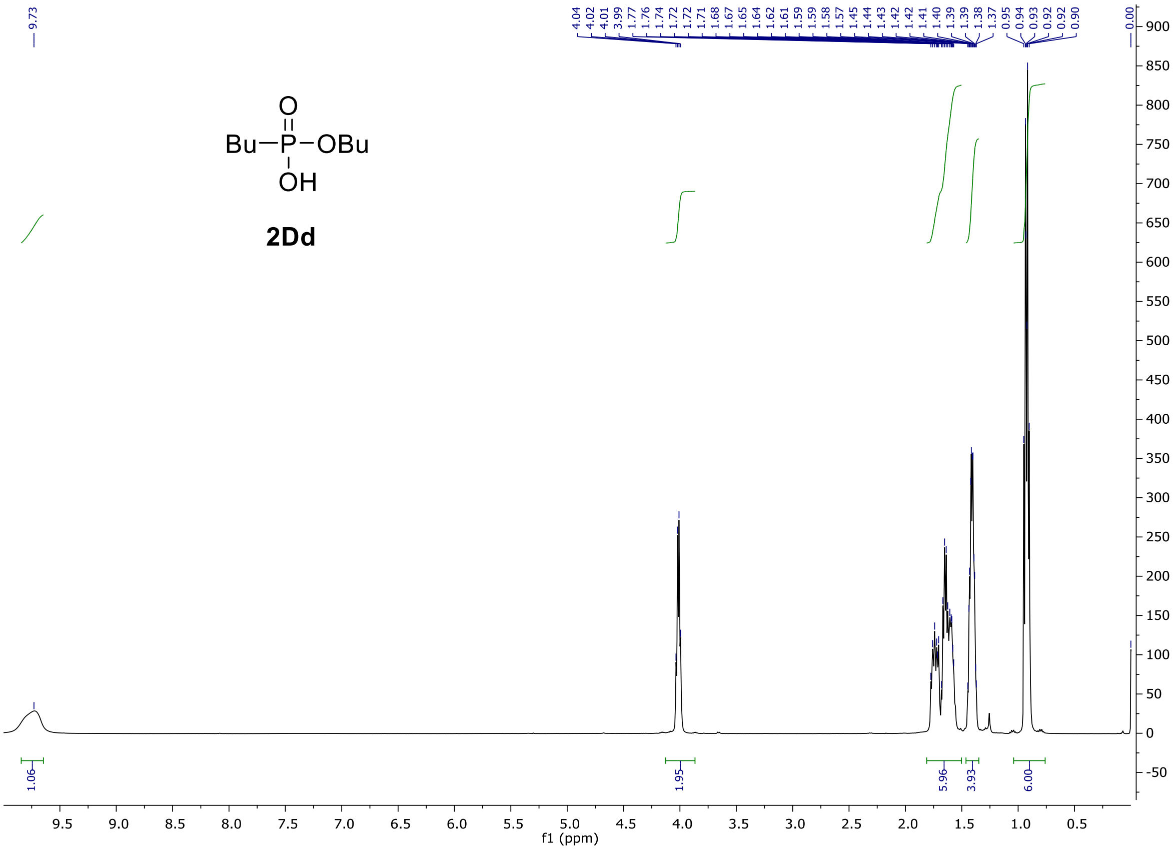
Bu−P(=O)(OH)−OBu
2Dd
f1 (ppm)

2. Table S1–S4 containing the computed row data

Table S1. Computed energies (*E*), zero point energies, internal energies (*U*), enthalpies (*H*) and Gibbs free energies (*G*) given in Hartree as well as entropies (*S*) given in J mol^{-1} K^{-1} at M06-2X/6-311+G(d,p) basis set considering a PCM solvent method using the parameter set of BuOH for **small molecules and ions**.

Compound	Nr.	E	ZPE	U	H	G	S	S_{vib}
**MeOH**		-115,71301	-115,661119	-115,66	-115,65692	-115,683805	56,585	1,268
**EtOH**		-155,02286	-154,94272	-154,938364	-154,93742	-154,968188	64,757	5,044
**BuOH**		-233,63117	-233,493521	-233,486596	-233,485652	-233,523788	80,265	15,514
**H_2O**		-76,433754	-76,412438	-76,409601	-76,408657	-76,430085	–	45,099
**$MeP(O)(OH)_2$**	**1A**	-608,22188	-608,150352	-608,143778	-608,142834	-608,180003	78,23	12,744
**$EtP(O)(OH)_2$**	**1B**	-647,52532	-647,425412	-647,41733	-647,416385	-647,45784	87,248	20,124
**$BuP(O)(OH)_2$**	**1D**	-726,13446	-725,977483	-725,967675	-725,96673	-726,012717	96,787	26,679
**$PhP(O)(OH)_2$**		-799,9271446	-799,801487	-799,791923	-799,790979	-799,836731	96,294	25,395
**MeP(O)(OH)OMe**		-647,50485	-647,404019	-647,396074	-647,39513	-647,435828	85,656	18,623
**MeP(O)(OH)OBu**	**2Ad**	-765,42355	-765,238128	-765,226807	-765,225863	-765,275649	104,784	33,573
**EtP(O)(OH)OBu**	**2Bd**	-804,72701	-804,513129	-804,499345	-804,498401	-804,555005	119,134	47,093
**BuP(O)(OH)OBu**	**2Dd**	-883,33566	-883,063926	-883,047887	-883,046942	-883,10861	129,79	55,668
**PhP(O)(OH)OMe**		-839,2103688	-839,056187	-839,045055	-839,044111	-839,094331	105,696	33,934
**PhP(O)(OH)OBu**		-957,1295201	-956,888737	-956,873955	-956,873011	-956,931856	123,849	49,21
**BF_4^-**		-424,634	-424,62	-424,616	-424,615	-424,648	69,373	4,742

Table S2. Computed energies (E), zero point energies, internal energies (U), enthalpies (H) and Gibbs free energies (G) given in Hartree as well as entropies (S) given in J mol^{-1} K^{-1} at M06-2X/6-311+G(d,p) basis set considering a SMD solvent method using the parameter set of BuOH for the monoesterification of $R^1P(O)(OH)_2$ (R^1 = Me, Et, Bu) with R^2OH (R^2 = Me, Bu).

R^1	R^2	Nr.	E	ZPE	U	H	G	S	S_{vib}	Imaginary frequency
Me	Me	**4**	-1563,637297	-1563,330057	-1563,304274	-1563,30333	-1563,386261	174,542	97,908	–
		**TS1**	-1563,606576	-1563,300781	-1563,277314	-1563,27637	-1563,352597	160,433	84,219	-408,97
		**5**	-1563,60872	-1563,300019	-1563,275367	-1563,274423	-1563,353609	166,662	90,398	–
		**TS2**	-1563,5671	-1563,2634	-1563,23864	-1563,2377	-1563,31796	168,919	92,59	-114,65
		**6**	-1563,6353	-1563,32861	-1563,30192	-1563,30097	-1563,38732	181,72	105,255	–
	Bu	**4**	-1681,5635	-1681,17113	-1681,14201	-1681,14107	-1681,23242	192,277	114,416	–
		**TS1**	-1681,5242	-1681,13372	-1681,10597	-1681,10503	-1681,19173	182,478	104,754	-429,69
		**5**	-1681,527	-1681,13301	-1681,10464	-1681,10369	-1681,19255	187,017	109,367	–
		**TS2**	-1681,4861	-1681,09644	-1681,06798	-1681,06704	-1681,15591	187,049	109,117	-134,50
		**6**	-1681,555	-1681,16223	-1681,13198	-1681,13103	-1681,22535	198,494	120,46	–
Et	Bu	**4**	-1760,170715	-1759,718843	-1759,687235	-1759,686291	-1759,783319	204,212	125,383	–
		**TS1**	-1760,130351	-1759,681528	-1759,65146	-1759,650516	-1759,74248	193,554	114,894	-466.98
		**5**	-1760,13309	-1759,680318	-1759,649263	-1759,648319	-1759,742886	199,032	120,349	–
		**TS2**	-1760,105478	-1759,657313	-1759,626012	-1759,625068	-1759,721756	203,498	124,861	-1182.16
		**6**	-1760,16366	-1759,712782	-1759,679838	-1759,678894	-1759,778817	210,304	131,674	–
Bu	Bu	**4**	-1917,3895	-1916,82387	-1916,78671	-1916,78577	-1916,89844	237,134	156,725	–
		**TS1**	-1917.3489	-1916.78546	-1916.75006	-1916.74912	-1916.8544	221.596	141.345	-344.09
		**5**	-1917,3521	-1916,78541	-1916,74881	-1916,74786	-1916,85648	228,612	148,288	–
		**TS2**	-1917,3189	-1916,75691	-1916,71991	-1916,71897	-1916,82887	231,316	150,988	-1029.18
		**6**	-1917,3815	-1916,81704	-1916,77855	-1916,77761	-1916,89271	242,246	161,835	–

Table S3. Computed energies (*E*), zero point energies, internal energies (*U*), enthalpies (*H*) and Gibbs free energies (*G*) given in Hartree as well as entropies (*S*) given in J mol^{-1} K^{-1} at M06-2X/6-311+G(d,p) basis set considering a SMD solvent method using the parameter set of BuOH for the monoesterification of $PhP(O)(OH)_2$ with R^2OH (R^2 = Me, Bu).

**R^2**	**Nr.**	**E**	**ZPE**	**U**	**H**	**G**	**S**	**S_{vib}**	**Imaginary frequency**
Me	**4**	-1947,055493	-1946,64034	-1946,6086	-1946,60765	-1946,70736	209,843	129,81	–
	**TS1**	-1947,021027	-1946,60819	-1946,57826	-1946,57732	-1946,67081	196,765	116,845	-455.44
	**5**	-1947,02296	-1946,60689	-1946,575928	-1946,574984	-1946,67209	204,377	124,447	–
	**TS2**	-1946.97491	-1946.56521	-1946.53547	-1946.53453	-1946.62706	194.761	104.753	-231.2
	**6**	-1947,046926	-1946,6326	-1946,59964	-1946,5987	-1946,70262	218,711	138,589	–

Table S4. Computed energies (*E*), zero point energies, internal energies (*U*), enthalpies (*H*) and Gibbs free energies (*G*) given in Hartree as well as entropies (*S*) given in J mol^{-1} K^{-1} at M06-2X/6-311+G(d,p) basis set considering a SMD solvent method using the parameter set of BuOH for the monoesterification of $R^1P(O)(OH)_2$ (R^1 = Me, Et) with R^2OH (R^2 = Me, Bu) in the presence of BF_4^-.

R^1	R^2	Nr.	E	ZPE	U	H	G	S	S_{vib}	Imaginary frequency
**Me**	**Me**	**7**	-1264,31	-1264,12	-1264,1	-1264,1	-1264,17	154,382	79,317	–
		**TS3**	-1264,24	-1264,05	-1264,03	-1264,03	-1264,1	146,116	71,008	-480.83
		**8**	-1264,31	-1264,12	-1264,1	-1264,1	-1264,17	161,4	85,904	–
	**Bu**	**7**	-1382,23	-1381,95	-1381,92	-1381,92	-1382	173,166	96,253	–
		**TS3**	-1382,16	-1381,88	-1381,86	-1381,86	-1381,94	164,409	87,311	-462.55
		**8**	-1382,23	-1381,95	-1381,93	-1381,93	-1382,01	180,346	102,912	–
**Et**	**Bu**	**7**	-1421,53	-1421,22	-1421,2	-1421,2	-1421,28	179,211	101,824	–
		**TS3**	-1421,47	-1421,16	-1421,13	-1421,13	-1421,21	169,304	92,089	-261.46
		**9**	-1421,53	-1421,22	-1421,19	-1421,19	-1421,28	189,363	111,849	–

3. XYZ geometries of computed species

4 (R^1 = Me, R^2 = Me)

P,0,1.3259280005,-2.5330835252,0.222284876
O,0,1.8394350046,-3.3525018118,-1.0595136942
O,0,2.4561756796,-1.6849363823,0.7104291386
O,0,0.0693264079,-1.72194417,-0.307843111
C,0,0.7269810238,-3.6974511508,1.4341626877
H,0,-0.069609106,-4.3115812964,1.0106280643
H,0,1.5558874441,-4.3338181635,1.7485943875
H,0,0.3495466458,-3.1473153877,2.2986398372
O,0,3.7522159168,0.2082531717,-0.6497986622
C,0,3.6738856768,-0.3698190244,-1.9540090956
H,0,4.0906111788,0.3476995805,-2.6609900806
H,0,4.2564995954,-1.2930585829,-1.9934861619
H,0,2.6361448625,-0.5808891988,-2.2283422348
C,0,-2.2019867893,1.3372207209,2.9766039213
H,0,-2.6114666691,1.6725486881,2.0177413433
H,0,-1.7679236657,2.194851663,3.4977770378
H,0,-3.0099995665,0.9275941962,3.5832238069
O,0,-1.2272114349,0.3132117835,2.7983325212
O,0,-1.9297336789,-1.602702495,1.1356663584
H,0,-1.6659688053,-0.9538927368,1.8414909712
C,0,-3.0871631734,-1.0563030807,0.4750653279
H,0,-3.5755964709,-1.8866193595,-0.0393752069
H,0,-3.7707420838,-0.6825543282,1.2440121553
C,0,-2.7119676468,0.0431785939,-0.5023049966
H,0,-2.1019707204,0.7919793154,0.0195462119
H,0,-2.0887748154,-0.3785326814,-1.2984396177
C,0,-3.9404821787,0.710782964,-1.1138925027
H,0,-4.5678643361,-0.0519747063,-1.5884382307
H,0,-4.5430068389,1.1605438244,-0.3168989499
C,0,-3.5625061692,1.7748244623,-2.139853342
H,0,-2.9851968381,1.3380601499,-2.9599539604
H,0,-4.449423557,2.2478921655,-2.5683593629
H,0,-2.9504271329,2.5589135086,-1.6844491569
P,0,0.835888041,1.8542093389,-0.1593574263
O,0,0.4228287685,0.8013709575,-1.2933742195
O,0,2.3089350122,2.3061376724,-0.518169657
O,0,0.6987044418,1.2695922627,1.2174034724
C,0,-0.1586603765,3.3027931958,-0.4440947434
H,0,-0.1286874282,3.5867847275,-1.4967182323
H,0,-1.1856455512,3.0850573601,-0.1454203291
H,0,0.2278872392,4.1198043815,0.1683203582
H,0,-0.4886662934,0.6813721982,2.2656078639
H,0,2.95759411,1.5190835982,-0.5557810085
H,0,3.3348158313,-0.4334493025,-0.0321290645
H,0,1.1477035288,-3.8142695134,-1.5548080485
H,0,-0.7971511719,-1.6954180878,0.3099310242
H,0,0.2809930891,-0.1128104955,-0.9577362694

TS1 (R^1 = Me, R^2 = Me)

P,0,0.3633081262,-1.93761375,-0.0158772446
O,0,1.6514761154,-2.7442318075,-0.7293846337
O,0,1.2025949144,-1.1387640616,1.0726524561
O,0,0.0108521243,-1.1951640505,-1.405365323
C,0,-0.3932403541,-3.5070209666,0.4565929173
H,0,-0.8303386051,-3.9612772767,-0.4355302021
H,0,0.4120855638,-4.1549389505,0.8027573721
H,0,-1.1468734797,-3.3916244458,1.2300412353
O,0,3.6005519936,-0.4943341929,0.4761441577
C,0,4.5569928649,-1.2406440831,-0.2701674305
H,0,5.5209578351,-0.7249337856,-0.277813219
H,0,4.6773537261,-2.2130571243,0.2082934955
H,0,4.2272893661,-1.3884443673,-1.3026888315
C,0,-1.6580831266,1.6356096209,2.5983923537
H,0,-1.8928315845,2.3228031428,1.7823651623
H,0,-1.3908893977,2.2031368209,3.4896540477
H,0,-2.5197265234,1.0038284196,2.8128919072
O,0,-0.5556725575,0.788251937,2.247835518
O,0,-1.0741946375,-1.0147936235,0.754107672
H,0,-0.8315267887,-0.0747452343,1.5345705084
C,0,-2.4306424047,-1.239024682,0.3621706874
H,0,-2.4696979586,-2.1113781566,-0.2977854239
H,0,-3.0054919669,-1.4803378492,1.2629051794
C,0,-3.0364572848,-0.037704276,-0.3459019276
H,0,-2.8928951678,0.8573646351,0.2705064973
H,0,-2.5146643323,0.1312580818,-1.2944831469
C,0,-4.525763694,-0.2425283745,-0.6141370379
H,0,-4.6631953248,-1.1591611159,-1.1984083658
H,0,-5.0462918859,-0.3959470673,0.3373847978
C,0,-5.1460674728,0.9372461323,-1.3560739691
H,0,-4.6564802028,1.0908718069,-2.3219803179
H,0,-6.2105746614,0.7759096216,-1.5421443623
H,0,-5.0436848315,1.8602805893,-0.7778393913
P,0,1.2321294609,2.1385372392,-0.4296910142
O,0,-0.1886390644,1.537475256,-1.0095447055
O,0,2.3074724471,1.2640226442,-1.0277556139
O,0,1.11940892,2.1805101159,1.094031404
C,0,1.3120885549,3.81319056,-1.0668208804
H,0,1.3114973382,3.7926905578,-2.1575693918
H,0,0.4604495347,4.3932199335,-0.7071496873
H,0,2.2355427561,4.2775920539,-0.7150869649
H,0,0.2002070898,1.3534135942,1.7876851022
H,0,3.2490955325,0.2402909639,-0.0821038759
H,0,2.1881161478,-1.0609165132,0.8694867588
H,0,2.1053397258,-2.2058357112,-1.38994652
H,0,-0.3354931207,-0.2905963744,-1.2736160561
H,0,-0.9731267095,1.9918081138,-0.6690036927

----

5 (R^1 = Me, R^2 = Me)

P,0,0.2243636965,-1.9014523019,-0.1503987409
O,0,1.5412653467,-2.657595927,-0.9315701059
O,0,1.1386211112,-1.1278682942,0.9165950949

O,0,-0.1375746865,-1.1608166464,-1.5572551738
C,0,-0.428269638,-3.5407701407,0.264305152
H,0,-0.8223335837,-4.0013759707,-0.64424807
H,0,0.406848828,-4.149535571,0.6110630991
H,0,-1.2026331901,-3.4957232192,1.0259155219
O,0,3.6323942929,-0.6209986108,0.2902693076
C,0,4.5719303408,-1.4045737065,-0.4371802899
H,0,5.547086567,-0.9101490217,-0.4614476553
H,0,4.6720473376,-2.3643131192,0.0703123657
H,0,4.2391461456,-1.5799034568,-1.4642283838
C,0,-1.4369066073,1.6659725332,2.6664030562
H,0,-1.6927229513,2.3090845318,1.8180615355
H,0,-1.1321687572,2.2943981117,3.5034866373
H,0,-2.3136981356,1.0850505389,2.9596312359
O,0,-0.3473482447,0.7991554441,2.3560070649
O,0,-1.1138369287,-1.0674019393,0.6385522948
H,0,-0.6507609621,0.0746086044,1.7508789162
C,0,-2.4625560964,-1.319102932,0.2509872576
H,0,-2.5008746795,-2.0817041227,-0.5365533809
H,0,-2.9995140857,-1.7179652039,1.1193686409
C,0,-3.1383520157,-0.0479473049,-0.2390043885
H,0,-3.011667305,0.7340567316,0.5189194768
H,0,-2.6420840963,0.3029569673,-1.1520240509
C,0,-4.6240391541,-0.2622021689,-0.5169499091
H,0,-4.7423617921,-1.0557298505,-1.2629184943
H,0,-5.1137588276,-0.6159645181,0.3967724051
C,0,-5.3108376956,1.0089309154,-1.0072896755
H,0,-4.8545675866,1.366774202,-1.9347974711
H,0,-6.372747282,0.8395073556,-1.2011902685
H,0,-5.2298490185,1.8075506101,-0.2641752744
P,0,1.2731645063,2.0962703213,-0.460865067
O,0,-0.2127074323,1.5684274941,-0.8401328083
O,0,2.2652116466,1.2235376653,-1.1645357982
O,0,1.3597436369,2.0891175694,1.1084108359
C,0,1.3647908789,3.809889175,-0.948262185
H,0,1.2849841579,3.8808660384,-2.0337307345
H,0,0.5611299297,4.3774202623,-0.4766142786
H,0,2.3274465615,4.2137642877,-0.6289639255
H,0,0.6819324322,1.4947604823,1.6451771949
H,0,3.3141635049,0.1165253466,-0.2684377942
H,0,2.1092383934,-1.1450097504,0.7089170722
H,0,1.9727289823,-2.0679857342,-1.5620473014
H,0,-0.5925628332,-0.3164499174,-1.4065091642
H,0,-0.9431097111,2.0070852401,-0.3779827755

TS2 (R^1 = Me, R^2 = Me)

P,0,1.9114057345,-0.1929319211,-0.194053866
O,0,2.615178081,2.362593862,-0.5704775677
O,0,1.2762235023,0.3987685042,1.052525809
O,0,1.2231288718,0.162291492,-1.5856151408
C,0,3.6869741294,-0.1687544394,-0.3423999975
H,0,3.9641468781,-0.1607369526,-1.3971401728
H,0,4.0730023202,0.7250218847,0.1379843211
H,0,4.0764007083,-1.0694164263,0.1369955284
O,0,-0.281073004,2.283139356,1.1328374365
C,0,0.0563641581,3.6780461198,0.9685995776
H,0,-0.7360146239,4.2584975637,1.4374633232
H,0,1.004811933,3.8517366305,1.4701023084
H,0,0.1338609331,3.9198751264,-0.0904494511
C,0,-0.480152054,-2.5615169287,2.183798894
H,0,-0.5796528741,-2.962854419,1.1724475792
H,0,-1.2431829629,-2.9986962245,2.8273382938
H,0,0.5076706031,-2.8025556013,2.5776773877
O,0,-0.6808743515,-1.1434093315,2.1932709284
O,0,1.5339784432,-1.7621908912,-0.0081227876
H,0,0.0996622848,-0.685088153,1.8143306685
C,0,1.8492786689,-2.7174458969,-1.027905825
H,0,2.9264058621,-2.7530092982,-1.2117509197
H,0,1.5165521197,-3.6864995175,-0.6597124482
P,0,-2.4357695209,0.0248827413,-0.5715855225
O,0,-1.3301461805,-0.8889695425,-1.3241917464
O,0,-1.8671268256,1.4206645478,-0.5463692862
O,0,-2.6874672619,-0.6228496401,0.8287917008
C,0,-3.9633519565,-0.1007807995,-1.4652464225
H,0,-3.8221145758,0.311636829,-2.4652344088
H,0,-4.267119115,-1.1466552597,-1.5310780782
H,0,-4.727332678,0.4713673064,-0.9360603013
H,0,-1.8445529186,-0.7997680909,1.4291872281
H,0,-0.9827391145,1.9519558208,0.4309452755
H,0,0.5150662141,1.6005733552,1.0745545563
H,0,1.9333860269,2.8991204477,-0.9856690078
H,0,0.3108093322,-0.1904930406,-1.6452732033
H,0,-1.5052430522,-1.8425774407,-1.3433819221
H,0,1.325586265,-2.4774957724,-1.9560037409
----

6 (R^1 = Me, R^2 = Me)

P,0,1.8418428291,-0.4050119783,-0.0239685899
O,0,2.6041188897,2.9659299807,-0.4782234328
O,0,1.3802026683,0.1646839327,1.278256349
O,0,1.3613927627,0.3969320036,-1.3210789918
C,0,3.6051115978,-0.5020568409,-0.2296137712
H,0,3.8506661471,-0.8875879152,-1.2204404427
H,0,4.0298249078,0.4959501975,-0.1085277105
H,0,4.0080504933,-1.1630295131,0.5398868522
O,0,0.0654926933,2.9844514962,0.6500851199
C,0,-0.5477858717,3.9739867957,-0.1685567568
H,0,-1.5474943213,4.2259822157,0.1970198864
H,0,0.0759598724,4.8682539051,-0.131521028
H,0,-0.6214781291,3.6390540109,-1.2085304962
C,0,-0.1546060344,-2.2406863045,3.0250021078
H,0,0.1782840602,-2.9032249736,2.2219653788
H,0,-0.9527177623,-2.7251779267,3.5866825042
H,0,0.6806725669,-2.0300071306,3.6953541233
O,0,-0.6766793344,-1.019029114,2.4954053649
O,0,1.2138000026,-1.872603181,-0.1638234856
H,0,0.0698679406,-0.5077882642,2.1039766554
C,0,1.4109064876,-2.6670627578,-1.3477336397
H,0,2.4384948641,-3.0339215233,-1.3842404817

H,0,0.723735356,-3.5074296961,-1.2718657693
P,0,-2.2937714077,-0.164958492,-0.4299661846
O,0,-1.2318462367,-0.4988299448,-1.6173529381
O,0,-1.9096629706,1.1655770017,0.1295772331
O,0,-2.2276599182,-1.3676553341,0.579004461
C,0,-3.9089230812,-0.2563209993,-1.1751074967
H,0,-3.9814330061,0.49242289,-1.9651336628
H,0,-4.0650296988,-1.2522679086,-1.5935495844
H,0,-4.6625227834,-0.0593299234,-0.4109635611
H,0,-1.585250004,-1.2335140036,1.3919109453
H,0,-0.4882180591,2.1853479421,0.5905038095
H,0,1.7618714034,2.8872714108,0.009108413
H,0,2.489362543,2.3651221782,-1.2225402723
H,0,0.4047292304,0.2957875262,-1.5116995324
H,0,-1.2496262527,-1.4112258101,-1.9448187001
H,0,1.1876625554,-2.084572952,-2.2439646748
----

4 (R[1] = Me, R[2] = Bu)

P,0,1.3259280005,-2.5330835252,0.222284876
O,0,1.8394350046,-3.3525018118,-1.0595136942
O,0,2.4561756796,-1.6849363823,0.7104291386
O,0,0.0693264079,-1.72194417,-0.307843111
C,0,0.7269810238,-3.6974511508,1.4341626877
H,0,-0.069609106,-4.3115812964,1.0106280643
H,0,1.5558874441,-4.3338181635,1.7485943875
H,0,0.3495466458,-3.1473153877,2.2986398372
O,0,3.7522159168,0.2082531717,-0.6497986622
C,0,3.6738856768,-0.3698190244,-1.9540090956
H,0,4.0906111788,0.3476995805,-2.6609900806
H,0,4.2564995954,-1.2930585829,-1.9934861619
H,0,2.6361448625,-0.5808891988,-2.2283422348
C,0,-2.2019867893,1.3372207209,2.9766039213
H,0,-2.6114666691,1.6725486881,2.0177413433
H,0,-1.7679236657,2.194851663,3.4977770378
H,0,-3.0099995665,0.9275941962,3.5832238069
O,0,-1.2272114349,0.3132117835,2.7983325212
O,0,-1.9297336789,-1.602702495,1.1356663584
H,0,-1.6659688053,-0.9538927368,1.8414909712
C,0,-3.0871631734,-1.0563030807,0.4750653279
H,0,-3.5755964709,-1.8866193595,-0.0393752069
H,0,-3.7707420838,-0.6825543282,1.2440121553
C,0,-2.7119676468,0.0431785939,-0.5023049966
H,0,-2.1019707204,0.7919793154,0.0195462119
H,0,-2.0887748154,-0.3785326814,-1.2984396177
C,0,-3.9404821787,0.710782964,-1.1138925027
H,0,-4.5678643361,-0.0519747063,-15884382307
H,0,-4.5430068389,1.1605438244,-0.3168989499
C,0,-3.5625061692,1.7748244623,-2.139853342
H,0,-2.9851968381,1.3380601499,-2.9599539604
H,0,-4.449423557,2.2478921655,-2.5683593629
H,0,-2.9504271329,2.5589135086,-1.6844491569
P,0,0.835888041,1.8542093389,-0.1593574263
O,0,0.4228287685,0.8013709575,-1.2933742195
O,0,2.3089350122,2.3061376724,-0.518169657
O,0,0.6987044418,1.2695922627,1.2174034724
C,0,-0.1586603765,3.3027931958,-0.4440947434
H,0,-0.1286874282,3.5867847275,-1.4967182323
H,0,-1.1856455512,3.0850573601,-0.1454203291
H,0,0.2278872392,4.1198043815,0.1683203582
H,0,-0.4886662934,0.6813721982,2.2656078639
H,0,2.95759411,1.5190835982,-0.5557810085
H,0,3.3348158313,-0.4334493025,-0.0321290645
H,0,1.1477035288,-3.8142695134,-1.5548080485
H,0,-0.7971511719,-1.6954180878,0.3099310242
H,0,0.2809930891,-0.1128104955,-0.9577362694

TS1 (R[1] = Me, R[2] = Bu)

P,0,0.3633081262,-1.93761375,-0.0158772446
O,0,1.6514761154,-2.7442318075,-0.7293846337
O,0,1.2025949144,-1.1387640616,1.0726524561
O,0,0.0108521243,-1.1951640505,-1.405365323
C,0,-0.3932403541,-3.5070209666,0.4565929173
H,0,-0.8303386051,-3.9612772767,-0.4355302021
H,0,0.4120855638,-4.1549389505,0.8027573721
H,0,-1.1468734797,-3.3916244458,1.2300412353
O,0,3.6005519936,-0.4943341929,0.4761441577
C,0,4.5569928649,-1.2406440831,-0.2701674305
H,0,5.5209578351,-0.7249337856,-0.277813219
H,0,4.6773537261,-2.2130571243,0.2082934955
H,0,4.2272893661,-1.3884443673,-1.3026888315
C,0,-1.6580831266,1.6356096209,2.5983923537
H,0,-1.8928315845,2.3228031428,1.7823651623
H,0,-1.3908893977,2.2031368209,3.4896540477
H,0,-2.5197265234,1.0038284196,2.8128919072
O,0,-0.5556725575,0.788251937,2.247835518
O,0,-1.0741946375,-1.0147936235,0.754107672
H,0,-0.8315267887,-0.0747452343,1.5345705084
C,0,-2.4306424047,-1.239024682,0.3621706874
H,0,-2.4696979586,-2.1113781566,-0.2977854239
H,0,-3.0054919669,-1.4803378492,1.2629051794
C,0,-3.0364572848,-0.037704276,-0.3459019276
H,0,-2.8928951678,0.8573646351,0.2705064973
H,0,-2.5146643323,0.1312580818,-1.2944831469
C,0,-4.525763694,-0.2425283745,-0.6141370379
H,0,-4.6631953248,-1.1591611159,-1.1984083658
H,0,-5.0462918859,-0.3959470673,0.3373847978
C,0,-5.1460674728,0.9372461323,-1.3560739691
H,0,-4.6564802028,1.0908718069,-2.3219803179
H,0,-6.2105746614,0.7759096216,-1.5421443623
H,0,-5.0436848315,1.8602805893,-0.7778393913
P,0,1.2321294609,2.1385372392,-0.4296910142
O,0,-0.1886390644,1.537475256,-1.0095447055
O,0,2.3074724471,1.2640226442,-1.0277556139
O,0,1.11940892,2.1805101159,1.094031404
C,0,1.3120885549,3.81319056,-1.0668208804
H,0,1.3114973382,3.7926905578,-2.1575693918
H,0,0.4604495347,4.3932199335,-0.7071496873
H,0,2.2355427561,4.2775920539,-0.7150869649

H,0,0.2002070898,1.3534135942,1.7876851022
H,0,3.2490955325,0.2402909639,-0.0821038759
H,0,2.1881161478,-1.0609165132,0.8694867588
H,0,2.1053397258,-2.2058357112,-1.38994652
H,0,-0.3354931207,-0.2905963744,-1.2736160561
H,0,-0.9731267095,1.9918081138,-0.6690036927
----

5 (R^1 = Me, R^2 = Bu)

P,0,1.8091928645,-1.1802877671,-0.2926729944
O,0,1.2023611502,-0.0709978628,-1.3025269322
O,0,1.8369113425,-0.0304107096,1.030739342
O,0,1.7755740342,-2.3114773228,-1.5670985019
C,0,3.5921120225,-1.4404627563,-0.0931339293
H,0,3.9702716042,-2.2323345589,-0.7335169942
H,0,4.0872704886,-0.4971794261,-0.342038588
H,0,3.7970908709,-1.6580241602,0.955220223
O,0,2.5391028878,2.5667611196,0.3480497483
C,0,3.1845115799,2.7510120988,-0.9083936591
H,0,3.0309054336,3.7668982816,-1.2837463138
H,0,4.2523568688,2.5904623306,-0.7575988619
H,0,2.8186334367,2.0364689584,-1.6519618893
C,0,-0.7067524867,-0.8785618189,3.1503508319
H,0,-1.3274161114,-1.5139428977,2.5130453119
H,0,-1.285756528,-0.5727123769,4.0225530318
H,0,0.1685470897,-1.4396590547,3.4815321085
O,0,-0.2964089595,0.3007983402,2.4595925516
O,0,0.7985590794,-2.0105914297,0.6714103505
H,0,0.5166984359,0.1266781416,1.9015889426
C,0,-0.2675278718,-2.8658890831,0.2322779426
H,0,0.1113635269,-3.6169385612,-0.4632549759
H,0,-0.5994384456,-3.3735803534,1.1406481107
C,0,-1.4016228893,-2.054304161,-0.3710822422
H,0,-1.6109277146,-1.2024544503,0.28867615
H,0,-1.0884854101,-1.6344205719,-1.333634626
C,0,-2.6664660564,-2.8844432418,-0.5650707685
H,0,-2.4424073201,-3.7481473745,-1.2008823965
H,0,-2.9913706598,-3.2824339754,0.4025699137
C,0,-3.7925018909,-2.063806547,-1.1869684029
H,0,-3.4937707721,-1.6678822006,-2.1620295268
H,0,-4.6934443921,-2.6651618297,-1.3306845207
H,0,-4.0559801621,-1.2176383586,-0.5442521357
P,0,-1.5429318534,2.1026847801,-0.2165429124
O,0,-2.1774462818,1.1421504518,-1.3424720555
O,0,-0.0555553163,2.1275038506,-0.4468533397
O,0,-1.9853329705,1.5385742021,1.1766640436
C,0,-2.3215511673,3.6962123408,-0.3854761154
H,0,-2.13666815,4.0775740194,-1.3906456024
H,0,-3.3960765771,3.6044158206,-0.2182400287
H,0,-1.8960061201,4.3801243286,0.3508227145
H,0,-1.2643612922,0.9798799507,1.7239822784
H,0,1.5809730355,2.6505048926,0.2025910737
H,0,2.3002870791,0.8037587533,0.8241664598
H,0,0.8181990052,0.7350256394,-0.8821638752
H,0,1.0165332713,-2.205122263,-2.1521801463
H,0,-3.0928857084,0.8707998122,-1.1798317942

TS2 (R^1 = Me, R^2 = Bu)

P,0,1.9239508238,-0.1945810547,-0.1917093109
O,0,2.7406707512,2.3012796897,-0.5353860789
O,0,1.3246496085,0.4062443922,1.0701218178
O,0,1.2476254742,0.2276322854,-1.5722136423
C,0,3.7008946541,-0.2697273685,-0.3280713904
H,0,3.9855504453,-0.2389496629,-1.3802173239
H,0,4.1347550935,0.5812464556,0.1882281743
H,0,4.0343801396,-1.2074660366,0.1211525343
O,0,-0.2962208977,2.2404061992,1.1172245454
C,0,0.045405633,3.6347667227,0.9568395422
H,0,-0.7847561664,4.219128831,1.3488064327
H,0,0.9478083664,3.8214660288,1.5336521362
H,0,0.2094920797,3.8599747766,-0.0961009072
C,0,-0.4194327655,-2.3773036351,2.483764221
H,0,-0.5415680305,-2.9241342286,1.5461718978
H,0,-1.1422950862,-2.7384402655,3.2149736138
H,0,0.5908300252,-2.5281874844,2.8662429919
O,0,-0.6674974949,-0.979503491,2.2891636046
O,0,1.4578219502,-1.7455158549,-0.0448821171
H,0,0.1171846331,-0.5561594632,1.8764265728
C,0,1.7560634642,-2.7139094222,-1.0696118252
H,0,2.8400658987,-2.7624323113,-1.2192966056
P,0,-2.3813259819,-0.0562145634,-0.6125378871
O,0,-1.2570128031,-0.9398545536,-1.3728388234
O,0,-1.8446604331,1.352607948,-0.5827404406
O,0,-2.6197375052,-0.7129801549,0.7853431773
C,0,-3.9057040108,-0.2191464779,-1.5061078395
H,0,-3.7725860787,0.1907380706,-2.5082860919
H,0,-4.1855978301,-1.2719904867,-1.566879426
H,0,-4.6816606223,0.3392170424,-0.9797256174
H,0,-1.7954222398,-0.7877850626,1.4340204646
H,0,-0.9812942572,1.9026709348,0.400804287
H,0,0.5104215805,1.5672915193,1.0768729437
H,0,2.0506062268,2.8344904815,-0.9410517677
H,0,0.3629152027,-0.1797297851,-1.6737443454
H,0,-1.4136748653,-1.8962572534,-1.394136448
H,0,1.288732196,-2.3989575538,-2.0086101632
C,0,1.2208544797,-4.056226931,-0.6194313028
H,0,1.6500201009,-4.2967466591,0.3594467778
H,0,0.1350868795,-3.9850094077,-0.4907391318
C,0,1.5520790794,-5.1608933262,-1.6199346408
H,0,2.6384578798,-5.2232123451,-1.7432588168
H,0,1.1389930029,-4.8998014797,-2.6002992041
C,0,1.0073165485,-6.5153092993,-1.1774096922
H,0,1.2500724784,-7.2969449249,-1.9010754232
H,0,1.4294177243,-6.8088408019,-0.2120432351
H,0,-0.0807510513,-6.4824074524,-1.0705889966
----

6 (R^1 = Me, R^2 = Bu)

P,0,1.9196892436,-0.4145849674,0.0766921981
O,0,2.8767390336,2.9391579765,0.4388107769
O,0,1.4933860548,-0.1293129083,1.4815506126
O,0,1.5365101485,0.7117243435,-0.9920795297
C,0,3.6721400059,-0.6054868215,-0.1606651987
H,0,3.8901374993,-0.8466499747,-1.2025556445
H,0,4.165316693,0.3311279462,0.1083988365
H,0,4.0317882901,-1.401142034,0.4933483356
O,0,0.118690415,2.9777886038,0.8438301099
C,0,-0.2762688075,3.6305114434,-0.3560907626
H,0,-1.3299549966,3.9229749706,-0.318406375
H,0,0.3318743047,4.5300955483,-0.4618868897
H,0,-0.1146174301,2.9959033282,-1.2343813124
C,0,-0.0969303691,-2.9377811323,2.5145302125
H,0,0.485906641,-3.3099398816,1.6683508914
H,0,-0.9473622375,-3.5970016294,2.6869983302
H,0,0.5275123556,-2.9178640216,3.4099560864
O,0,-0.6130370139,-1.6325032052,2.2445310875
O,0,1.1609854977,-1.7396777513,-0.3989797431
H,0,0.1417855231,-1.0254170372,2.0730511537
C,0,1.2169755888,-2.2411175258,-1.7558649183
H,0,0.1812697471,-2.4349524151,-2.0441348668
P,0,-2.1880626249,0.0739860972,-0.2701986204
O,0,-1.134329852,0.2962624548,-1.4909988131
O,0,-1.8593633596,1.0884466853,0.7753762377
O,0,-2.0380386418,-1.4232342085,0.189997818
C,0,-3.8085600612,0.1948941955,-0.9941202252
H,0,-3.9363390294,1.1907182126,-1.4212429727
H,0,-3.9266939114,-0.5595037559,-1.7741449022
H,0,-4.5554491363,0.030584418,-0.2157574491
H,0,-1.4476987329,-1.5512996185,1.0346226071
H,0,-0.4725592348,2.2118324745,0.9595927204
H,0,1.9471772399,2.8630106512,0.7231843087
H,0,2.8786181035,2.5048792093,-0.4211055275
H,0,0.5727289257,0.7636750486,-1.1705490963
H,0,-1.1763392539,-0.3595447221,-2.2038311469
H,0,1.6238240756,-1.4721988147,-2.4198190699
C,0,2.0464534929,-3.5075236301,-1.8045174438
H,0,3.057304612,-3.2863657329,-1.444007834
H,0,1.6078888551,-4.2429017587,-1.1218359066
C,0,2.1111718839,-4.0813598097,-3.2180110075
H,0,2.5446247458,-3.3351304364,-3.8927558097
H,0,1.0952810444,-4.2789677997,-3.5769603578
C,0,2.9336256657,-5.3645971881,-3.2779864907
H,0,2.972151963,-5.7642627656,-4.2940439807
H,0,3.9611527175,-5.1861451812,-2.9486480047
H,0,2.5039935164,-6.1344034002,-2.6307641138

----

4 (R^1 = Et, R^2 = Bu)

P,0,1.0772722985,-2.2429114717,0.1322244941
O,0,1.7754484879,-3.388869556,-0.7459169372
O,0,2.1226035911,-1.5840183658,0.9745855298
O,0,0.4257769819,-1.3031752618,-0.9757362151
C,0,-0.2692641437,-2.9688481238,1.0677461763
H,0,0.1849486384,-3.648673031,1.7939120762
H,0,-0.7234859871,-2.149527322,1.6346048985
O,0,3.573828068,0.1482625624,-0.5201017335
C,0,3.9428440333,-0.7430751676,-1.5746465724
H,0,4.6019924176,-0.1982340171,-2.2503047069
H,0,4.4780749642,-1.6063679745,-1.1737877175
H,0,3.0630726958,-1.0817130355,-2.1298370173
C,0,-1.3926540381,1.7866048563,2.9024232731
H,0,-1.5808577751,2.404666055,2.0173371725
H,0,-0.865919281,2.3834956962,3.6513079756
H,0,-2.3480288757,1.4614014385,3.3154677134
O,0,-0.6429184218,0.6198857448,2.5651016418
O,0,-1.5103287547,0.061742007,0.138956423
H,0,-1.2489279134,0.1455925562,1.0932523778
C,0,-2.8693616232,-0.3933036763,0.0436607001
H,0,-2.9652019943,-1.3397972359,0.5870636787
H,0,-3.5223298436,0.3447134737,0.520863363
C,0,-3.2270547694,-0.5688424102,-1.4169372302
H,0,-3.155703176,0.402160416,-1.9201213397
H,0,-2.4903708253,-1.2317478511,-1.8863822359
C,0,-4.6292090424,-1.1460377697,-1.5919010175
H,0,-4.6790947273,-2.125617071,-1.1035115475
H,0,-5.352830751,-0.5022531124,-1.0799659128
C,0,-5.0167383858,-1.2850406212,-3.060819994
H,0,-4.3215065807,-1.9444027965,-3.5885136924
H,0,-6.0204108402,-1.7030885974,-3.1688530778
H,0,-5.0016911679,-0.3129527519,-3.5618873773
P,0,1.0503010195,2.4834739729,-0.1627650856
O,0,-0.4614783955,2.3949101362,-0.6702648109
O,0,1.9448271602,1.9120992372,-1.3389837593
O,0,1.2903605387,1.7526804501,1.1302432462
C,0,1.348060909,4.2458437464,-0.0831756197
H,0,1.1452892297,4.6608308932,-1.0741794511
H,0,2.4178539885,4.364139083,0.1104127217
H,0,0.1964027074,0.9354049901,2.1650725776
H,0,2.6158947753,1.2070634016,-1.0226581531
H,0,3.0875330896,-0.3752800359,0.1533209331
H,0,1.2350152181,-3.7656633879,-1.4556380828
H,0,-0.3359042626,-0.7550904259,-0.6267312277
H,0,-0.9222154223,1.5429027993,-0.4659580965
C,0,-1.2949376082,-3.6896007803,0.1916804632
H,0,-2.1145779241,-4.0627555003,0.8077722686
H,0,-1.7144746433,-3.0233806523,-0.5658570328
H,0,-0.8428160573,-4.5443931932,-0.3175685435
C,0,0.5097588054,4.9279079094,0.9976106362
H,0,0.771668503,5.9855744011,1.0593113885
H,0,-0.5570186396,4.8569500178,0.7755711798
H,0,0.6894998197,4.477522622,1.9765171991

TS1 (R^1 = Et, R^2 = Bu)

P,0,0.6289672133,1.6483730459,-0.0066445856
O,0,-0.1555859505,3.1049907752,-0.2867690288

O,0,-0.2380889347,1.2279996472,1.2647076533
O,0,0.2196828615,1.0905429936,-1.463278669
C,0,2.218806151,2.5013099681,0.223928927
H,0,2.0128945465,3.2392474316,1.0046748375
H,0,2.9596190498,1.8055978322,0.6109260467
O,0,-2.6776097224,2.0572405775,1.2881769836
C,0,-3.2124029328,3.3211353171,0.9084215799
H,0,-4.2713577538,3.3833621661,1.1739186756
H,0,-2.6658769325,4.0980494504,1.4439352753
H,0,-3.1089061547,3.4893849651,-0.1676706764
C,0,0.682800403,-2.9516283235,1.3872015895
H,0,0.9689836088,-3.0498878477,0.3361743418
H,0,-0.1495628263,-3.6209302903,1.6052526713
H,0,1.528053102,-3.2043352454,2.0257140273
O,0,0.2743796952,-1.6052580009,1.6737918797
O,0,1.4593655134,0.0013370929,0.3404131821
H,0,0.8554299938,-0.8268195646,1.0385154183
C,0,2.4763396807,-0.5086726548,-0.5257279781
H,0,2.8401809988,0.3049201897,-1.161269487
P,0,-2.409686601,-1.1531202944,-0.4310107686
O,0,-1.022444867,-1.3601211127,-1.2991519513
O,0,-2.8382396269,0.2713163203,-0.689982387
O,0,-2.0967181875,-1.5333372007,1.0174492942
C,0,-3.5721353339,-2.3399635218,-1.1296092812
H,0,-0.7460059257,-1.5125092913,1.4362762433
H,0,-2.8926233409,1.3913289632,0.5947891027
H,0,-1.1096326967,1.720340324,1.3392058559
H,0,-0.9659129961,2.9989894435,-0.8003701354
H,0,0.0567547607,0.127218627,-1.4934194504
H,0,-0.6419705606,-2.2471367859,-1.2230946408
H,0,2.0369658354,-1.2658409508,-1.1870523452
C,0,2.7174388342,3.2058854738,-1.040089276
H,0,3.6600748915,3.7103032864,-0.818248345
H,0,2.8974134638,2.5004958313,-1.8542118415
H,0,2.0041245422,3.9536043432,-1.3867853684
C,0,3.623321039,-1.0911187142,0.2795949654
H,0,4.0392332744,-0.3053371761,0.9203536179
H,0,3.2403050827,-1.875162477,0.9423428466
C,0,4.7147367,-1.6633470352,-0.6210642956
H,0,4.2830530855,-2.4328785184,-1.2705542464
H,0,5.0901856512,-0.8728101483,-1.2800970771
C,0,5.8700169096,-2.2572586657,0.178623499
H,0,5.5221462191,-3.0714461261,0.8210411426
H,0,6.3309736496,-1.5003758,0.8197856507
C,0,-4.9342452381,-2.2750194232,-0.4367163864
H,0,-5.3833217715,-1.2853957061,-0.5470630843
H,0,-5.6162837631,-3.0054072524,-0.8758180423
H,0,-4.8449639938,-2.4930463666,0.6297952678
H,0,-3.6625517958,-2.1167496953,-2.1960870176
H,0,-3.1294468099,-3.3354509487,-1.0307749416
H,0,6.6454769597,-2.6573609275,-0.4789362681
----

5 (R^1 = Et, R^2 = Bu)

P,0,1.706263223,0.6691263382,-0.4249647604
O,0,1.6365290539,2.3675144644,-0.6373069229
O,0,1.0134077818,0.850804779,1.0140194923
O,0,0.8485763456,0.4583823421,-1.7864606747
C,0,3.5241972775,0.6275473009,-0.5486177164
H,0,3.830614888,1.3733835786,-1.2835742865
H,0,3.8619968203,-0.3510200005,-0.8860576178
O,0,-0.7903083822,2.76354088,1.3157839672
C,0,-0.801985215,4.0712836144,0.753926854
H,0,-1.7416786161,4.5824171148,0.9837787912
H,0,0.0223917365,4.6342089405,1.1931105341
H,0,-0.6675628805,4.0365716749,-0.3304404259
C,0,-0.1783920287,-3.1955321347,1.7103423527
H,0,-0.1841376039,-3.5051520482,0.6607211441
H,0,-1.0970976351,-3.537040306,2.1883962509
H,0,0.675733598,-3.6461440235,2.2196110907
O,0,-0.1289685846,-1.7725700044,1.8244027305
O,0,1.6167154465,-1.051244916,-0.0410834864
H,0,0.5643075373,-1.4300686996,1.2042426285
C,0,1.9941568619,-2.0533334563,-0.9801383981
H,0,2.4623855667,-1.6017057727,-1.8627612285
P,0,-2.3948079894,-0.3173415655,-0.3809923868
O,0,-1.3544150424,-1.177719411,-1.2886784912
O,0,-1.991323959,1.1200844282,-0.4745446803
O,0,-2.3421387597,-0.95310847,1.0565066871
C,0,-4.0464891501,-0.6573942272,-0.9869651961
H,0,-4.079632381,-0.3015667925,-2.02005443
H,0,-4.1792373843,-1.7426114078,-0.9999718062
H,0,-1.3997692109,-1.2296913004,1.4143875003
H,0,-1.3466357175,2.1863144889,0.7544547727
H,0,0.5589921632,1.7222486065,1.1266524169
H,0,1.8085751262,2.6318095892,-1.5480731876
H,0,0.277393338,-0.3299764431,-1.7663244947
H,0,-1.4822536073,-2.1380463057,-1.2587411508
H,0,1.087694489,-2.5659479149,-1.3293038001
C,0,-5.1181897489,0.0225848234,-0.1322391107
H,0,-6.1078879223,-0.2006080039,-0.5337839025
H,0,-5.08253,-0.330695183,0.9000675398
H,0,-4.9903752466,1.1070650345,-0.1302147183
C,0,4.1340849295,0.9477719798,0.820020647
H,0,5.2232336837,0.947344075,0.7457603788
H,0,3.8178572401,1.9311510925,1.1746271486
H,0,3.8439945587,0.2029646498,1.5647382954
C,0,2.9490082382,-3.0447975486,-0.3388812223
H,0,2.4830063474,-3.4550842405,0.5637817051
H,0,3.8509015959,-2.5104840039,-0.0171607683
C,0,3.3238168051,-4.1780269554,-1.2901894172
H,0,2.4157547961,-4.7115806228,-1.5926307282
H,0,3.7576766554,-3.7564592376,-2.2038637745
C,0,4.3093818442,-5.1600197209,-0.6640845732
H,0,3.8923865729,-5.6092571012,0.2420080896
H,0,4.5568685424,-5.9698823147,-1.3549269186
H,0,5.2410805133,-4.6573974131,-0.3889393024

TS2 (R^1 = Et, R^2 = Bu)

P,0,0.8192933422,1.569599776,0.2564812765
O,0,-0.0274605687,3.4236793411,0.5062660055
O,0,0.1483851837,1.5427912324,1.6950733444
O,0,-0.0413461322,1.4977096802,-1.0902818367
C,0,2.400785719,2.4078686641,0.0297292912
H,0,2.2186604578,3.3451338399,-0.492049726
H,0,3.0147616328,1.7712357969,-0.6116304061
O,0,-2.6980092477,2.7848861467,-0.3401330577
C,0,-3.0521104331,2.6254646906,-1.706594558
H,0,-4.0085541844,2.1024725132,-1.8103010934
H,0,-3.1498793848,3.6200047257,-2.1442001478
H,0,-2.2830361123,2.0735711942,-2.2565322615
C,0,0.8736827181,-1.5663057068,2.9880442985
H,0,1.2840858133,-2.0346165137,2.0900817886
H,0,0.6965043742,-2.3267460579,3.7486322899
H,0,1.5849508965,-0.8315693136,3.3697791159
O,0,-0.3791264369,-0.93669194,2.6982676426
O,0,1.2917467757,-0.0069693114,0.358912571
H,0,-0.2080278252,-0.0278473188,2.3730483982
C,0,1.9869511383,-0.694164312,-0.6915638404
H,0,2.05716972,-0.0650428471,-1.5865367144
P,0,-2.2662768453,-1.2111127109,-0.2350595614
O,0,-1.2770853608,-0.9097920396,-1.4925437615
O,0,-2.7017177464,0.1102552624,0.3098425421
O,0,-1.4512013958,-2.1079611215,0.7683258067
C,0,-3.5738414184,-2.2563658251,-0.8675261911
H,0,-4.0377174041,-1.7113200201,-1.6942276122
H,0,-3.1058074417,-3.1550436558,-1.2795596111
H,0,-1.0243671981,-1.6126073435,1.5774432837
H,0,-2.6175665444,1.8871178486,0.029074156
H,0,-0.1861279172,2.6213397399,1.5161534543
H,0,-0.8843513872,3.4287763597,0.045856794
H,0,-0.3675639633,0.5989198669,-1.309698477
H,0,-0.8301251933,-1.6836053225,-1.8683576383
H,0,1.3865564216,-1.5721864364,-0.9496179606
C,0,-4.597695081,-2.6046054217,0.2140906058
H,0,-5.3792600947,-3.2384784016,-0.2072431716
H,0,-4.1306286167,-3.1440946417,1.0405878283
H,0,-5.0684197392,-1.7037104093,0.6128207124
C,0,3.0842507576,2.6402977196,1.3785107224
H,0,4.0663138104,3.0884454704,1.218207589
H,0,2.4978957919,3.3166336504,2.0036030924
H,0,3.2228403837,1.7020863209,1.9219480027
C,0,3.3665123131,-1.1156899923,-0.221316277
H,0,3.2594523271,-1.7409911988,0.6717406152
H,0,3.9349040188,-0.2265460485,0.07481111
C,0,4.1224377592,-1.8795241184,-1.3053365045
H,0,3.540667071,-2.759011005,-1.6026814695
H,0,4.2127399847,-1.2481518103,-2.1962249097
C,0,5.509469137,-2.315022223,-0.8430594249
H,0,5.4423831126,-2.9746880966,0.0268262622
H,0,6.0421132968,-2.8523130443,-1.6314060809
H,0,6.1147817154,-1.4492496311,-0.5592763061
----

6 (R^1 = Et, R^2 = Bu)

P,0,1.7112970487,-0.5695071152,-0.0233667513
O,0,3.0503539367,2.5174721529,0.452012138
O,0,1.2642514582,-0.2028276403,1.35685175
O,0,1.4349797229,0.5365658788,-1.1454330832
C,0,3.4524189823,-0.9348234153,-0.19800266
H,0,3.6491766876,-1.1315496651,-1.2556551518
H,0,3.9944515978,-0.0272206043,0.0797332885
O,0,0.3097379703,2.8694520427,0.7014394773
C,0,-0.0710153075,3.5612233301,-0.4800882667
H,0,-0.9865996921,4.1396706536,-0.3214015362
H,0,0.7360766714,4.2483951999,-0.7398931379
H,0,-0.229354256,2.8737542677,-1.3185658606
C,0,-0.3210833516,-2.9654405527,2.4401272223
H,0,0.1693555376,-3.329837585,1.5342578188
H,0,-1.1703687431,-3.6060481905,2.6766388967
H,0,0.3849280977,-2.9835903583,3.2730516554
O,0,-0.8216636583,-1.6396188737,2.2515512086
O,0,0.8761002413,-1.8641727735,-0.4694790471
H,0,-0.065594256,-1.0504383728,2.0266866645
C,0,1.0272910265,-2.447937385,-1.7827154585
H,0,2.0045792503,-2.9383938382,-1.8346622659
P,0,-2.3299656067,0.1683125818,-0.2505200449
O,0,-1.2808396633,0.1252574605,-1.4958440731
O,0,-1.8375381341,1.2130444585,0.6980227127
O,0,-2.3958074155,-1.2900618183,0.3347177225
C,0,-3.9378614916,0.4831107808,-0.9695991525
H,0,-4.174219884,-0.3551954643,-1.630895932
H,0,-4.6538103562,0.4630131288,-0.1433259767
H,0,-1.7493994311,-1.4731108551,1.1288403147
H,0,-0.3628738196,2.1803533985,0.8559084062
H,0,2.0927905675,2.5407658343,0.6406817314
H,0,3.0928092374,2.2571668483,-0.4739442315
H,0,0.4787789294,0.6619961421,-1.3193735421
H,0,-1.402571923,-0.6189326013,-2.1063773071
H,0,0.9920606511,-1.6553120444,-2.5368767513
C,0,-0.0934517134,-3.44352493,-1.984857921
H,0,-0.0502860389,-4.1938437254,-1.1883683536
H,0,-1.0538517446,-2.9244270141,-1.8891322701
C,0,0.0015487318,-4.1228732421,-3.3488297377
H,0,0.965891755,-4.6352898948,-3.4308276227
H,0,-0.0178294573,-3.3622787103,-4.1368828225
C,0,-1.1305380973,-5.1209522723,-3.570542188
H,0,-1.0494892666,-5.6053120886,-4.5464299212
H,0,-1.1165920871,-5.9023713666,-2.8054864058
H,0,-2.1042379051,-4.6249038765,-3.5225160435
C,0,3.855460535,-2.1196050785,0.6817289704
H,0,3.3132040527,-3.0258461484,0.4004234096
H,0,4.9226854445,-2.3156623859,0.5704672326
H,0,3.6561761866,-1.9126420482,1.7351547609
C,0,-3.9798975412,1.8187964422,-1.7115500887
H,0,-4.9806116442,1.9907955435,-2.1105391415
H,0,-3.7354674765,2.6474978866,-1.0434888068
H,0,-3.2755422784,1.8312565337,-2.5460742577
----

4 (R^1 = Bu, R^2 = Bu)

P,0,-2.1441832722,-1.6158945142,0.1280887943
O,0,-3.2101107963,-2.490967275,-0.6904155598
O,0,-1.2864996612,-2.5377732885,0.9356623343
O,0,-1.374908914,-0.837713031,-1.027500478
C,0,-3.0282557833,-0.3947955363,1.1005487012
H,0,-3.5602082146,-0.9449528918,1.883040522
H,0,-2.2656251844,0.2188229198,1.5948977787
O,0,0.5194044163,-3.8058174294,-0.6443386033
C,0,-0.3733397928,-4.2908196975,-1.6498036616
H,0,0.2210509806,-4.8419735592,-2.3783288886
H,0,-1.1135031921,-4.962197724,-1.2094011121
H,0,-0.8814156347,-3.463554652,-2.1539755717
C,0,1.7582172848,1.432722914,2.5968678666
H,0,2.2431983031,1.7034652149,1.6523110989
H,0,2.498858608,0.9927906163,3.2692976497
H,0,1.3525734192,2.3339609108,3.0575683383
O,0,0.6742703543,0.5296282864,2.3780494048
O,0,-0.2384856839,1.2569853177,0.0233082729
H,0,-0.0239409561,1.0321038286,0.9680026706
C,0,-0.9533250884,2.5007691403,-0.0378615477
H,0,-1.8557062173,2.4193963012,0.5786131662
H,0,-0.3209852417,3.2921725164,0.3775004625
C,0,-1.3148961548,2.7954309247,-1.4780649833
H,0,-0.3958593831,2.8976544583,-2.0660980023
H,0,-1.8670410433,1.9411228574,-1.8878415248
C,0,-2.1580758918,4.0620367078,-1.5955784228
H,0,-3.0500784505,3.9593676132,-0.9667381673
H,0,-1.5931461103,4.9121439644,-1.1983470995
C,0,-2.5760679236,4.3492898223,-3.0341601024
H,0,-3.1714609238,3.5261237892,-3.4399448309
H,0,-3.175211823,5.2606183654,-3.0993356043
H,0,-1.7008806006,4.4769463625,-3.6776167457
P,0,2.5015772523,-0.9646819787,-0.4911241305
O,0,2.127053045,0.4954748358,-1.0224765422
O,0,1.9976450372,-1.9810380192,-1.5965553244
O,0,1.9218604853,-1.2744508354,0.862176782
C,0,4.2896475331,-0.9621457166,-0.5571304874
H,0,4.5820551871,-0.7497701932,-1.5904883616
H,0,4.6102683314,-1.9828040442,-0.3251248864
H,0,1.0593530402,-0.2700406273,1.9587214157
H,0,1.4082847339,-2.7283158309,-1.2173907127
H,0,-0.0251487263,-3.3816186064,0.0537843731
H,0,-3.6954649631,-2.0162965442,-1.3812000296
H,0,-0.9167964643,-0.0092072586,-0.6962719403
H,0,1.2473654952,0.8412037264,-0.7358117687
C,0,-3.988608053,0.4661549864,0.2757724437
H,0,-3.4417091189,0.9616863471,-0.5350014842
H,0,-4.7412139294,-0.176637676,-0.1948716149
C,0,4.9100320601,0.0435698141,0.4160682581
H,0,4.5709940979,1.0536371839,0.1624154463
H,0,4.5587992895,-0.1670238008,1.4323082151
C,0,-4.6938965232,1.5178770754,1.1290175129
H,0,-3.9450475101,2.1775346378,1.581401588
H,0,-5.2136878364,1.0208214059,1.9549930053
C,0,-5.6865888496,2.3390596874,0.3131121504
H,0,-6.1743606426,3.1004263659,0.9265498896
H,0,-5.1883807019,2.8464656065,-0.5185362006
H,0,-6.4677993809,1.6997572886,-0.1080946622
C,0,6.4363903157,0.0013041531,0.3891752269
H,0,6.7837059019,0.1993619502,-0.6306741681
H,0,6.7732044206,-1.0087238587,0.6461178119
C,0,7.0543750745,1.0115441257,1.3505170468
H,0,8.1460118894,0.9749892395,1.3200474735
H,0,6.7466519697,2.030239058,1.097098247
H,0,6.7406791114,0.8143102698,2.379804273

TS1 (R^1 = Bu, R^2 = Bu)

P,0,-1.8705982193,-0.8241939078,0.2075897969
O,0,-2.4473994745,-2.2298544358,-0.5135712871
O,0,-0.9352301948,-1.548790189,1.2743857317
O,0,-1.2374181373,-0.3191075133,-1.1887115985
C,0,-3.5532669964,-0.331767494,0.6814812732
H,0,-3.9690282598,-1.2233837957,1.1604047865
H,0,-3.5178845187,0.4638144609,1.4239185188
O,0,0.0506475945,-3.8192309658,0.6051799424
C,0,-0.5995537024,-4.9161464252,-0.0284258741
H,0,0.0580080599,-5.7894508888,-0.0490044132
H,0,-1.4925163049,-5.1584320988,0.5485619182
H,0,-0.8937109733,-4.6688249962,-1.0521457813
C,0,1.5360602825,1.5989932897,2.6537161153
H,0,2.1388942703,1.8566116625,1.7790383419
H,0,2.1898188311,1.4243701428,3.5080740385
H,0,0.845382228,2.4103358962,2.8822707802
O,0,0.7736735916,0.4085445661,2.4127039908
O,0,-1.1570063134,0.7150891561,1.0148760982
H,0,-0.1454183127,0.5780333239,1.7531541961
C,0,-1.5802472425,2.0431291437,0.7016235697
H,0,-2.5157253224,1.9985321985,0.1363841252
H,0,-1.7931326021,2.5558268318,1.6462551818
C,0,-0.5445436123,2.8201340385,-0.095971248
H,0,0.409278947,2.815726242,0.4445041985
H,0,-0.3767172258,2.3291079858,-1.0609790586
C,0,-0.9914003335,4.2610434898,-0.3333607043
H,0,-1.9540944984,4.2578019359,-0.8563476958
H,0,-1.1596683926,4.7515454335,0.6316515071
C,0,0.0287319152,5.0551039562,-1.1434070348
H,0,0.1954642619,4.5921914106,-2.1203399596
H,0,-0.3076540148,6.0809088492,-1.311709706
H,0,0.9907017261,5.0986492502,-0.6243793335
P,0,2.2310472989,-1.1106563913,-0.3409152728
O,0,1.441889864,0.2596686116,-0.8106316322
O,0,1.4887851549,-2.2447615974,-1.0059650868
O,0,2.2770149437,-1.1000347486,1.1888295963
C,0,3.9001927839,-0.9347041773,-0.9948122813
H,0,3.8142639048,-0.8791777444,-2.0846552945
H,0,4.4227708776,-1.867118195,-0.755229595
H,0,1.386943156,-0.297895237,1.9238112296
H,0,0.6673232658,-3.3807511558,-0.025640632
H,0,-0.7135544365,-2.5040189118,1.0481383877

H,0,-1.8544699571,-2.5579008719,-1.2013536246
H,0,-0.3933766079,0.1599886527,-1.0742039552
H,0,1.7613976016,1.0684695381,-0.3848878043
C,0,-4.4277306789,0.0589129487,-0.5166484923
H,0,-3.9799718363,0.8982085568,-1.0591753434
H,0,-4.4810456946,-0.7781903559,-1.2179578617
C,0,4.6636975204,0.2683991363,-0.4391007546
H,0,4.1184932442,1.1922116246,-0.667975725
H,0,4.7209894077,0.1929341549,0.652093826
C,0,-5.8359668499,0.4441053795,-0.0684706356
H,0,-5.7724787298,1.2755009281,0.6421131705
H,0,-6.2843396747,-0.3974777129,0.4704274248
C,0,-6.7232936728,0.8373028437,-1.2453192084
H,0,-7.7261086448,1.1156774919,-0.9125888231
H,0,-6.2999942972,1.6898752593,-1.7843583319
H,0,-6.8224578601,0.0098806496,-1.9537515546
C,0,6.074990837,0.3699508375,-1.0144239978
H,0,6.0136880009,0.4375068166,-2.1060254085
H,0,6.6207900334,-0.5519510707,-0.7864923816
C,0,6.8370270467,1.5712697383,-0.4643874338
H,0,7.843624891,1.6327536764,-0.8850377764
H,0,6.3179618265,2.5047189947,-0.7011725917
H,0,6.9319662258,1.5083357772,0.6236994482

5 (R^1 = Bu, R^2 = Bu)

P,0,-1.8174767119,-0.6471723355,0.1253692461
O,0,-2.3385768837,-2.1310612347,-0.544161757
O,0,-0.8176778565,-1.3564642771,1.1649709769
O,0,-1.2772572687,-0.2114629944,-1.3505275182
C,0,-3.5351095484,-0.290401878,0.6242951446
H,0,-3.8489403657,-1.1811728739,1.1772468481
H,0,-3.5479630697,0.5519421442,1.3156021015
O,0,0.0078499297,-3.8163811641,0.6397383291
C,0,-0.6793774463,-4.8757110368,-0.0165761228
H,0,-0.0740128555,-5.7868950137,-0.0162707463
H,0,-1.6058476805,-5.0671157792,0.5256966341
H,0,-0.9224617526,-4.6157555276,-1.0509573364
C,0,1.6764500768,1.6642098103,2.603258936
H,0,2.0897225767,2.0255191414,1.6558250758
H,0,2.4991709259,1.4179580242,3.2749122106
H,0,1.0691763771,2.4493298094,3.0583252963
O,0,0.9034331188,0.4806155005,2.4183907774
O,0,-1.1790230591,0.8502874103,0.810772913
H,0,0.1047947246,0.6657186799,1.8610912471
C,0,-1.7218238495,2.1171728216,0.4496088378
H,0,-2.4609026646,2.005690193,-0.3520574402
H,0,-2.2414208663,2.537610005,1.3188584991
C,0,-0.6216100278,3.0611533839,-0.0064128766
H,0,0.1370691819,3.1307943451,0.7812348295
H,0,-0.1313033044,2.6419219758,-0.8939428477
C,0,-1.1608029056,4.4533332789,-0.3254528631
H,0,-1.9536767095,4.3700145472,-1.0769535044
H,0,-1.6236220919,4.874842487,0.5734906628
C,0,-0.0727534747,5.3948312429,-0.8317874836
H,0,0.3799894904,5.0104828388,-1.7503317784
H,0,-0.4736713084,6.3882843294,-1.0467642889
H,0,0.7224955174,5.5092534266,-0.0892456414
P,0,2.2421368928,-1.1144284602,-0.408216254
O,0,1.5181777721,0.273779646,-0.8297949952
O,0,1.4609634303,-2.2405402583,-1.0126276084
O,0,2.3225375351,-1.1171724057,1.1626735104
C,0,3.939174481,-1.0464328939,-0.9820894391
H,0,3.9014670961,-1.0687318565,-2.0754719655
H,0,4.4030034897,-1.9823260307,-0.6518657569
H,0,1.6674955892,-0.502036138,1.6985438088
H,0,0.6457214935,-3.4113614286,0.017933426
H,0,-0.7105742444,-2.3284471413,1.0113303796
H,0,-1.704884121,-2.4734225029,-1.18664846
H,0,-0.5535200714,0.4310272568,-1.292525139
H,0,1.9043471931,1.0776978454,-0.4513301045
C,0,-4.4992676979,-0.0548835435,-0.5433323571
H,0,-4.1845902438,0.8156473521,-1.1289746879
H,0,-4.4759709307,-0.9160957058,-1.2158115333
C,0,4.7312883318,0.1616023747,-0.4787136169
H,0,4.2558671312,1.0863946442,-0.826273983
H,0,4.7147628605,0.1826902223,0.6161886212
C,0,-5.9271374443,0.1644621344,-0.0458823772
H,0,-5.9480938742,1.0294402097,0.6260451603
H,0,-6.2344555298,-0.7028207123,0.5487173582
C,0,-6.910559997,0.3779089849,-1.1919999723
H,0,-7.9274601081,0.5271097411,-0.8207422774
H,0,-6.6383940324,1.255974568,-1.7851004335
H,0,-6.9225165002,-0.4864823598,-1.8622133804
C,0,6.1793713449,0.132355543,-0.9631563973
H,0,6.1919387272,0.0984124432,-2.0577546276
H,0,6.6542018341,-0.7903976903,-0.6132203759
C,0,6.9723242446,1.3395167933,-0.4738968703
H,0,8.0066698469,1.3051895377,-0.8242494803
H,0,6.5284822763,2.2720673111,-0.8344677632
H,0,6.9904490066,1.3779272394,0.6191202306

TS2 (R^1 = Bu, R^2 = Bu)

P,0,-1.86645582,-0.281264246,0.4453050344
O,0,-2.4073935014,-2.2233136552,-0.2884843718
O,0,-1.0669641051,-1.3256674015,1.300993483
O,0,-1.41081962,0.0379455509,-1.0549411461
C,0,-3.6424240285,-0.0405258177,0.6574489518
H,0,-4.0096254823,-0.9483006978,1.1408650981
H,0,-3.7780791759,0.7925728519,1.3521713682
O,0,-0.0093209318,-4.1314258788,0.7044383674
C,0,-0.3716114236,-5.0450316544,-0.3186256651
H,0,0.501630378,-5.3714234656,-0.8938565757
H,0,-0.8211988233,-5.9185446071,0.1562779938
H,0,-1.1044231093,-4.6093503146,-1.0064820082
C,0,1.8615192419,1.4756500231,2.8367910574
H,0,2.2711001789,1.824415909,1.8834073798
H,0,2.6835193816,1.1738392047,3.4853506974
H,0,1.30628878,2.2853306526,3.3141139127
O,0,1.0203599686,0.3347660749,2.654889102

O,0,-1.230473075,1.0345946505,1.2144805663
H,0,0.1902575915,0.6038983631,2.2146058082
C,0,-1.5756741085,2.3776459496,0.819703629
H,0,-2.4459936273,2.3612383905,0.1555372825
H,0,-1.8557156572,2.9060254166,1.7340944235
C,0,-0.4039907588,3.0583203866,0.1392314562
H,0,0.477596419,2.9762007882,0.7857478723
H,0,-0.1750203382,2.5391931068,-0.7976016543
C,0,-0.6997496023,4.528135059,-0.1506051126
H,0,-1.6026920164,4.5994163623,-0.7668126805
H,0,-0.9182471933,5.0462977614,0.7891011565
C,0,0.4617789013,5.2160417385,-0.8612473971
H,0,0.6757310106,4.7300493251,-1.8174569243
H,0,0.2401110776,6.2668869231,-1.0618787736
H,0,1.3705441598,5.1754786682,-0.2536020677
P,0,2.0255483233,-1.1485001456,-0.3874460234
O,0,1.3047461601,0.2888794327,-0.6423555054
O,0,1.1166243625,-2.1978195547,-0.940874582
O,0,2.2939560498,-1.2260326293,1.1621278849
C,0,3.6398582536,-1.0845355679,-1.1582522168
H,0,3.4637901971,-0.9618368856,-2.2317608691
H,0,4.0871035614,-2.0735613788,-1.0112536318
H,0,1.704395924,-0.6543009698,1.7858398272
H,0,0.3958151579,-3.3647435495,0.2637673139
H,0,-1.5136450211,-2.1636277077,0.6947788945
H,0,-1.971377579,-2.3940967129,-1.1310201039
H,0,-0.4624146118,0.2792459624,-1.0997825033
H,0,1.7458397858,1.069132268,-0.2730297048
C,0,-4.3922982292,0.2091462254,-0.6532839412
H,0,-4.0238173852,1.1273949767,-1.1227093815
H,0,-4.1927406741,-0.6121135304,-1.3470396917
C,0,4.5524359435,0.0147951181,-0.6108100104
H,0,4.0723316093,0.9935683839,-0.7306679637
H,0,4.7064734384,-0.1393486518,0.462016542
C,0,-5.8956819456,0.3276855912,-0.4139051677
H,0,-6.0853079857,1.1296240726,0.3078854244
H,0,-6.2594492585,-0.5994287237,0.0417284312
C,0,-6.659030746,0.6040626768,-1.7053014032
H,0,-7.7336241698,0.6811119583,-1.5234182428
H,0,-6.3275919107,1.5412094312,-2.1618718937
H,0,-6.4985180012,-0.1970189565,-2.4325222007
C,0,5.9050726518,0.0326347532,-1.3197847269
H,0,5.7471004097,0.1900268987,-2.3920296062
H,0,6.3769157614,-0.9497160957,-1.2111596988
C,0,6.8279426999,1.1138191128,-0.7674190753
H,0,7.7900978373,1.1170795132,-1.2850707378
H,0,6.3820929417,2.1060308901,-0.8836851432
H,0,7.0206257586,0.9555293768,0.2975854435
----

6 (R^1 = Bu, R^2 = Bu)

P,0,1.7321041862,-0.5690399185,-0.1152850076
O,0,2.8237913363,2.7757762496,-0.0359683254
O,0,1.3051747985,-0.1417415101,1.253611185
O,0,1.4095361022,0.4776686444,-1.2838370976
C,0,3.480275188,-0.8892423776,-0.3023168564
H,0,3.6669758669,-1.1244343552,-1.3547172377
H,0,3.997541043,0.0484868415,-0.0744003812
O,0,0.3710823659,2.7638286676,1.2677444607
C,0,-0.2183459839,3.9490383321,0.7457519151
H,0,-1.2217752162,4.1029068521,1.1529929474
H,0,0.4125691605,4.7888221249,1.0403821481
H,0,-0.276967519,3.9187251295,-0.3469827396
C,0,-0.4912281021,-2.7542135715,2.3633075424
H,0,-0.2056289418,-3.2462903815,1.430523931
H,0,-1.330423565,-3.2836241126,2.8138578158
H,0,0.3522506968,-2.7650171324,3.0564421742
O,0,-0.9102587238,-1.4085975061,2.1195265482
O,0,0.913861669,-1.8970061695,-0.4815480058
H,0,-0.1192778065,-0.8752208,1.8770964471
C,0,1.0103512045,-2.5279775607,-1.7793907279
H,0,2.0325917682,-2.8941530257,-1.9181597201
P,0,-2.2713034655,0.1870205733,-0.5917913456
O,0,-1.2489176541,-0.050774628,-1.8382978814
O,0,-1.6823019145,1.2844052198,0.2334146879
O,0,-2.4175220584,-1.1983483197,0.1384587818
C,0,-3.872167488,0.5355068058,-1.3098465119
H,0,-4.1527980537,-0.3259895956,-1.9245785568
H,0,-4.5787792792,0.5884389818,-0.4752605171
H,0,-1.8000253423,-1.3101716903,0.9714760777
H,0,-0.1937678944,2.020045554,0.9932306603
H,0,2.0161419302,2.691443258,0.5066022182
H,0,2.6135827767,2.2681776804,-0.8279254119
H,0,0.4637537353,0.4978138861,-1.54374751
H,0,-1.4140368787,-0.8429491265,-2.3723652992
H,0,0.7931738462,-1.7856176573,-2.5538528954
C,0,0.0132687235,-3.6648596033,-1.8126858531
H,0,0.2648952478,-4.3773227946,-1.0198952328
H,0,-0.9834181225,-3.2675350729,-1.589842412
C,0,0.0085476113,-4.3708079088,-3.1663248215
H,0,1.0213984379,-4.7163404174,-3.3995574094
H,0,-0.2665814591,-3.6559231593,-3.9494536548
C,0,-0.9532644697,-5.5544411161,-3.1920433665
H,0,-0.9525315962,-6.0441101135,-4.1686179062
H,0,-0.6743309351,-6.3001176315,-2.4422631006
H,0,-1.9767113182,-5.2332280209,-2.9783161767
C,0,3.9511535373,-2.0203380906,0.6167760402
H,0,3.3906212652,-2.9353091388,0.3932878407
H,0,3.7339377043,-1.7594161964,1.6577996771
C,0,-3.8873466839,1.8308417838,-2.1249228861
H,0,-3.5898938144,2.6678668515,-1.4845105084
H,0,-3.1499007788,1.7680926191,-2.9325584981
C,0,-5.2671272747,2.1091132985,-2.7172668539
H,0,-5.5616395123,1.2686996515,-3.3552190033
H,0,-6.0013652617,2.1614448797,-1.9061175638
C,0,-5.2940494226,3.4037934912,-3.5226777521
H,0,-4.585594355,3.361175504,-4.3550870477
H,0,-6.2866002249,3.5950020039,-3.9373145302
H,0,-5.0222273096,4.2584899118,-2.8966558758
C,0,5.4457704251,-2.2897736136,0.4572149631
H,0,5.654172753,-2.5444433659,-0.5874868045

H,0,5.9997559947,-1.3704985451,0.6744502676
C,0,5.925890004,-3.412936348,1.3700145201
H,0,5.397486161,-4.3461080482,1.1534629461
H,0,6.9952511593,-3.59860071,1.2442612243
H,0,5.7502388071,-3.165775432,2.4208842758
----

4 (R^1 = Ph, R^2 = Me)

P,0,2.4848045132,1.5249079967,0.321366533
O,0,3.3386162277,2.8710721883,0.2292937014
O,0,1.6473630636,1.3960890717,1.5542114517
O,0,1.6313213905,1.5695462859,-1.0238517495
O,0,-0.6828913396,2.7417863235,1.3954997014
C,0,-0.1353583243,3.9989208985,0.9919808378
H,0,-0.9657741195,4.6887594824,0.8442362018
H,0,0.5220967686,4.3902773257,1.771441996
H,0,0.4187739489,3.9026388867,0.0528711125
C,0,-0.0280848426,-3.2428023132,0.9045114399
H,0,-0.6948571971,-3.1685228686,0.0383407034
H,0,-0.5888150468,-3.6397039592,1.7543594489
H,0,0.7887176782,-3.9252068965,0.6667865096
O,0,0.5436584001,-1.9738279724,1.2223556864
O,0,0.9276435585,-0.9595315321,-1.1704101479
H,0,0.9033034124,-1.3080388382,-0.2386787148
C,0,1.7944076788,-1.7895191725,-1.9528243121
H,0,2.658521383,-2.0992167862,-1.3596032244
H,0,1.2547392151,-2.6724060621,-2.3039736695
P,0,-2.2411613137,0.0496423842,-0.0983351703
O,0,-1.7246010312,-0.7713842767,-1.3630729701
O,0,-1.987409853,1.5735489938,-0.4165593784
O,0,-1.6137665962,-0.3881192997,1.1941851209
H,0,-0.1995366041,-1.3587472151,1.4007044514
H,0,-1.4743112818,2.0732049791,0.3234644938
H,0,0.0723215055,2.1441726591,1.5857640494
H,0,2.8162410465,3.6885867832,0.2151686826
H,0,1.2408081061,0.6725417337,-1.2086115613
H,0,-0.7366204344,-0.859029942,-1.4206531002
C,0,3.6974269801,0.2221849325,0.1571030864
C,0,4.5625041934,0.1917962927,-0.9406400295
C,0,3.7183943287,-0.8010417058,1.1046879286
C,0,5.451748141,-0.8663378571,-1.0823139069
H,0,4.5381643324,0.9860235464,-1.6799075961
C,0,4.6078325763,-1.8606558107,0.9527117272
H,0,3.0392143629,-0.770401705,1.9486367315
C,0,5.4713404852,-1.8917704529,-0.1380102726
H,0,6.1275681714,-0.8935540637,-1.9291494272
H,0,4.6252784419,-2.6582500144,1.6863171542
H,0,6.1633987697,-2.7183244647,-0.2547975904
C,0,-4.0131575056,-0.219716524,-0.1571370313
C,0,-4.4971058188,-1.5047657442,0.106703476
C,0,-4.8998743227,0.8200124353,-0.4345239234
C,0,-5.8650244351,-1.7454040942,0.0892325435
H,0,-3.8072781606,-2.3130496013,0.3292917298
C,0,-6.2701548745,0.5732547758,-0.4478344303
H,0,-4.5242057399,1.8161212093,-0.6376763923
C,0,-6.7510697763,-0.7058950557,-0.1879793324
H,0,-6.2401047847,-2.7412616791,0.2953363709
H,0,-6.9597842176,1.3815075286,-0.6619592348
H,0,-7.8185391228,-0.8954144411,-0.1992689133
H,0,2.1346410632,-1.2089353643,-2.8107387908

TS1 (R^1 = Ph, R^2 = Me)

P,0,1.8777004463,0.4557687615,-0.2303798759
O,0,2.183055745,2.0863054256,-0.4198478573
O,0,1.2098055158,0.5361610448,1.2066021688
O,0,0.9529800834,0.4273099622,-1.5489286827
O,0,-0.0828239669,2.6395399993,1.7829582662
C,0,0.2802857992,3.9984252874,1.5553817327
H,0,-0.4463845585,4.6691428828,2.0208196285
H,0,1.2587109675,4.1634094089,2.006898794
H,0,0.3376371563,4.2216432141,0.4867043753
C,0,-0.5277554705,-3.3772992301,1.3552984831
H,0,-0.8644996297,-3.4196524539,0.3163028032
H,0,-1.3607243491,-3.593624936,2.0236363615
H,0,0.2683027629,-4.1024543131,1.5167628232
O,0,-0.0157031694,-2.0717751647,1.6674911217
O,0,1.5381557624,-1.3664023296,-0.0125336463
H,0,0.7472710651,-1.7187253374,0.8648781789
C,0,1.7840023081,-2.3094420796,-1.0503798892
H,0,2.67202347,-2.0232495908,-1.619455217
H,0,1.9624805405,-3.2854697151,-0.595011492
P,0,-2.2278836471,0.1293285216,0.1694750122
O,0,-1.4618944901,-0.7579094896,-0.9908479738
O,0,-1.726247805,1.5367502832,-0.0157584186
O,0,-1.9860049362,-0.568678724,1.5012973576
H,0,-0.8077107684,-1.3975238235,1.6538527322
H,0,-0.8115562788,2.3739527832,1.1776843629
H,0,0.8561786113,1.4508690108,1.4619986653
H,0,1.4139439263,2.568517809,-0.7508812352
H,0,0.2060351098,-0.2025151674,-1.4965483083
H,0,-1.7417735491,-1.6844235026,-1.0235429883
C,0,3.6413350017,0.0499448267,-0.3416425357
C,0,4.4035247568,0.5611589991,-1.3915278402
C,0,4.2360040325,-0.7582582976,0.6281984277
C,0,5.756699747,0.248184643,-1.4801152017
H,0,3.9434004633,1.1973145659,-2.1391246442
C,0,5.5954048809,-1.0399377631,0.5528747837
H,0,3.6419016102,-1.1597829919,1.4412442738
C,0,6.3537919907,-0.5454387467,-0.5053403485
H,0,6.3442351786,0.632592061,-2.3059023608
H,0,6.0605091785,-1.6517161348,1.3173124766
H,0,7.4110674465,-0.7775028369,-0.5685517916
C,0,-3.9792090599,0.0069527505,-0.2510652113
C,0,-4.7591155912,-1.0335881945,0.257721625
C,0,-4.5495608252,0.9457055692,-1.1139204866
C,0,-6.0996692129,-1.1373551675,-0.1004447715
H,0,-4.3210551962,-1.756235355,0.9381264217

C,0,-5.8903905613,0.8402460152,-1.4682390277
H,0,-3.9456903931,1.7582883037,-1.5029476559
C,0,-6.6645679337,-0.2010202882,-0.9627789552
H,0,-6.7042204112,-1.944396135,0.2975430665
H,0,-6.3309829793,1.5717984313,-2.136085212
H,0,-7.7102137838,-0.2811564076,-1.2384819952
H,0,0.9300010102,-2.3742333836,-1.7317073197
----

5 (R[1] = Ph, R[2] = Me)

P,0,1.8611716957,0.3419780636,-0.2784099264
O,0,2.1190819491,2.0097932192,-0.4357293467
O,0,1.1245251054,0.4556459126,1.1403869564
O,0,0.9961088355,0.3677231446,-1.6549391134
O,0,-0.0627440529,2.6986677572,1.7662846415
C,0,0.3246482132,4.0454392224,1.513862833
H,0,-0.40133379,4.741842841,1.9422729664
H,0,1.2938327763,4.2070548386,1.9861602217
H,0,0.4155496671,4.2414364235,0.4417649102
C,0,-0.5840952385,-3.3949225584,1.3209660097
H,0,-0.816093811,-3.3864628531,0.2517188154
H,0,-1.4824358734,-3.6515499045,1.8827573865
H,0,0.1867651363,-4.1426659024,1.5176298107
O,0,-0.146343912,-2.1075635353,1.7614135044
O,0,1.5650892135,-1.378970058,-0.0960402094
H,0,0.5696211209,-1.7981148121,1.1493650022
C,0,1.920824029,-2.3023815176,-1.1122541188
H,0,2.8334368731,-2.0004510893,-1.6369374718
H,0,2.0993613938,-3.2717094097,-0.6421446087
P,0,-2.2216978724,0.1391773935,0.0831955266
O,0,-1.4657656027,-0.8192710526,-0.9873702163
O,0,-1.6830166515,1.522160762,-0.0815232182
O,0,-2.0268430474,-0.5324122201,1.4836819816
H,0,-1.1919311401,-1.1614965322,1.6255930861
H,0,-0.7879669854,2.4423222775,1.1640577581
H,0,0.8746839811,1.3823972783,1.3975234342
H,0,1.3334986723,2.4674691541,-0.7610385289
H,0,0.3334377946,-0.3422034997,-1.6888461184
H,0,-1.8210228466,-1.7182347952,-1.0581202034
C,0,3.660826974,0.0615464004,-0.3123606553
C,0,4.4516805797,0.6354374346,-1.3075046517
C,0,4.2486093319,-0.7445757612,0.6641487098
C,0,5.8217804054,0.3908332284,-1.3343634468
H,0,3.9989906264,1.2688176277,-2.0617640663
C,0,5.6230156261,-0.9577307918,0.6545051724
H,0,3.6349830763,-1.200019717,1.4335635524
C,0,6.4089933687,-0.3985144922,-0.3498581362
H,0,6.4302049506,0.8253072208,-2.1194005951
H,0,6.078368691,-1.5676075414,1.4264998194
H,0,7.4782823843,-0.5775221058,-0.3641907239
C,0,-3.9799107903,0.0277704064,-0.2548050107
C,0,-4.6981160732,-1.1081930354,0.1277481097
C,0,-4.618107649,1.0703420419,-0.9283826693
C,0,-6.0523351258,-1.2010632447,-0.1709349666
H,0,-4.2019113692,-1.9126491822,0.6611065319

C,0,-5.9750000865,0.9737972186,-1.2200690232
H,0,-4.0572069885,1.9527455355,-1.2157835147
C,0,-6.6891097949,-0.1603116314,-0.8439369386
H,0,-6.6120341756,-2.0809345998,0.1248381316
H,0,-6.4743307723,1.7845173621,-1.7379965996
H,0,-7.7466481063,-0.232753835,-1.0720740005
H,0,1.1148882844,-2.4089090864,-1.8469607932

TS2 (R[1] = Ph, R[2] = Me)

P	1.89998	-0.11727	-0.09919
O	2.32624	2.38389	-0.29636
O	1.41431	0.31481	1.30899
O	0.99318	0.20859	-1.34747
O	0.10333	2.25715	1.9023
C	0.45753	3.63202	2.07199
H	-0.22667	4.09762	2.78397
H	1.47253	3.66582	2.46466
H	0.4189	4.16667	1.12149
C	-0.36569	-3.00804	1.89235
H	-0.54578	-3.02431	0.81452
H	-1.17214	-3.53066	2.40594
H	0.58283	-3.49905	2.11312
O	-0.35554	-1.66603	2.39273
O	1.66825	-1.70026	0.14697
H	0.41675	-1.18717	2.04595
C	1.88453	-2.65177	-0.90462
H	2.89194	-2.55286	-1.31683
H	1.771	-3.63702	-0.45644
P	-2.37055	0.15719	0.2053
O	-1.44355	-0.81171	-0.71641
O	-1.75591	1.51616	0.14092
O	-2.44038	-0.53345	1.60976
H	-1.56363	-0.95588	1.97162
H	-0.66678	2.15188	1.2999
H	0.98397	1.27985	1.50007
H	1.44933	2.78089	-0.31887
H	0.10082	-0.20453	-1.2895
H	-1.72779	-1.73625	-0.78488
C	3.63071	-0.0146	-0.5088
C	4.04846	0.3726	-1.78078
C	4.55142	-0.42321	0.4556
C	5.40399	0.35199	-2.0854
H	3.32277	0.69608	-2.51733
C	5.90762	-0.42588	0.14472
H	4.21793	-0.7333	1.44063
C	6.33198	-0.04191	-1.12375
H	5.73641	0.65233	-3.07219
H	6.62903	-0.73229	0.89316
H	7.38915	-0.04881	-1.36436
C	-4.04084	0.09289	-0.42853
C	-4.88585	-0.96777	-0.09208
C	-4.48228	1.10598	-1.28185
C	-6.17279	-1.01292	-0.61464
H	-4.54366	-1.74694	0.58006
C	-5.77188	1.05415	-1.79955
H	-3.8249	1.93121	-1.53247

C -6.6142 -0.00328 -1.46651
H -6.83205 -1.83203 -0.35223
H -6.11978 1.84119 -2.45832
H -7.62039 -0.03934 -1.86872
H 1.14313 -2.51669 -1.69491

6 (R^1 = Ph, R^2 = Me)

P,0,1.860294,-0.606785,-0.114633
O,0,2.232777,3.010922,-0.311023
O,0,1.427237,-0.529952,1.310703
O,0,1.080933,0.342505,-1.1334
O,0,0.518532,2.468717,1.815666
C,0,-0.122291,3.728495,1.986327
H,0,-0.928334,3.664832,2.723099
H,0,0.626236,4.435201,2.347219
H,0,-0.529974,4.099842,1.040879
C,0,-0.332461,-3.294977,1.890848
H,0,-0.544462,-3.264115,0.818422
H,0,-1.052488,-3.947754,2.383306
H,0,0.675538,-3.679986,2.053214
O,0,-0.461866,-1.993475,2.472698
O,0,1.612087,-2.101315,-0.63144
H,0,0.256202,-1.421845,2.109626
C,0,1.887398,-2.483233,-1.992444
H,0,2.963214,-2.463808,-2.177411
H,0,1.513543,-3.498525,-2.10502
P,0,-2.347945,-0.016794,0.407539
O,0,-1.390403,-0.774567,-0.667058
O,0,-1.679845,1.281135,0.714869
O,0,-2.558402,-1.019829,1.589838
H,0,-1.679931,-1.406832,2.021325
H,0,-0.153318,1.85537,1.465749
H,0,1.702257,2.758851,0.468975
H,0,1.977048,2.36201,-0.97643
H,0,0.124956,0.133186,-1.180993
H,0,-1.689157,-1.64632,-0.967085
C,0,3.567508,-0.158639,-0.390513
C,0,4.005853,0.272438,-1.646243
C,0,4.463769,-0.263023,0.674616
C,0,5.344444,0.596456,-1.830796
H,0,3.304699,0.36469,-2.468481
C,0,5.80174,0.062486,0.480905
H,0,4.115236,-0.587331,1.649001
C,0,6.239695,0.491087,-0.768987
H,0,5.687547,0.93677,-2.800791
H,0,6.499683,-0.011987,1.306492
H,0,7.282516,0.748851,-0.916148
C,0,-3.965997,0.157746,-0.340489
C,0,-4.900975,-0.877486,-0.266948
C,0,-4.269665,1.335623,-1.026191
C,0,-6.138405,-0.731936,-0.883958
H,0,-4.666404,-1.786704,0.275115
C,0,-5.509403,1.473958,-1.640852
H,0,-3.543582,2.13991,-1.074424
C,0,-6.441109,0.441814,-1.569722
H,0,-6.867774,-1.531424,-0.823617
H,0,-5.748929,2.388127,-2.17134
H,0,-7.408333,0.554358,-2.046797
H,0,1.369771,-1.819662,-2.687614

----

7 (R^1 = Me, R^2 = Me)

P,0,0.5825630142,-1.4986224596,-0.0868895782
O,0,-0.6386691988,-2.5004047435,0.1810654172
O,0,0.504871032,-1.0941720519,-1.6376274648
O,0,0.5579059886,-0.3246938332,0.8379994131
C,0,2.0301908577,-2.5243452836,-0.0248692226
H,0,2.1705408021,-2.8704145688,1.0002407514
H,0,1.91401046,-3.3797633093,-0.6920069325
H,0,2.8904100076,-1.9284648926,-0.3354363057
C,0,0.8761432556,3.0839523519,0.491701128
H,0,1.5662876138,3.8133860201,0.0644442677
H,0,1.3227450107,2.6612316338,1.3970625567
H,0,-0.05420425,3.5933519212,0.7594367747
O,0,0.643903911,2.0749121701,-0.485020491
H,0,0.2819492911,1.2992430082,-0.0206683209
O,0,3.086065821,0.7838732192,-1.013552005
H,0,2.2299947113,1.2428547993,-0.9462961026
C,0,3.7105520903,0.8536673236,0.259625918
H,0,4.6490034521,0.2990464322,0.2002682106
H,0,3.9401938396,1.8878382071,0.5395427212
H,0,-0.231351753,-0.4917620536,-1.8347738571
H,0,-1.4300090232,-2.0392552875,0.5213237928
B,0,-2.5941780885,0.4200875471,0.2574034688
F,0,-2.5875313937,-0.7607266326,1.0467670541
F,0,-1.9747684807,1.457154697,0.9522527861
F,0,-3.9113572773,0.7554684193,-0.0541029009
F,0,-1.8810445524,0.1469822304,-0.9351591683
H,0,3.087497859,0.4041451354,1.0399210893

TS3 (R^1 = Me, R^2 = Me)

P,0,1.8003841285,-0.5098107618,0.240262868
O,0,1.1454674591,-2.700464628,0.537677724
O,0,0.3080953159,-0.6759871515,-0.2921134794
O,0,2.0982491838,-0.325711223,1.6938857502
C,0,3.0689714024,-1.2521378711,-0.7953571591
H,0,3.5571649164,-2.0555843275,-0.2492151474
H,0,2.5990074357,-1.656564871,-1.6929625854
H,0,3.7984715058,-0.4925690338,-1.0808503858
C,0,-0.7251392451,2.707565055,0.4707750624
H,0,-0.0947696914,3.5898608074,0.5520632722
H,0,-0.5761100586,2.0358756539,1.3153886002
H,0,-1.7697169714,2.9953302148,0.3806945137
O,0,-0.3426925815,2.0356141771,-0.7594081297
H,0,-0.978652615,1.3043871196,-0.9545826482
O,0,1.8625599294,1.0860084501,-0.4072003175
H,0,0.6553491631,1.6065901245,-0.6454758285
C,0,2.8408975386,2.0093360449,0.0933681197
H,0,3.8379513637,1.5696919305,0.0239301627

H,0,2.7943866059,2.8982930157,-0.5347510957
H,0,0.2209856489,-1.6814336763,-0.0234468177
H,0,0.9886625487,-2.8400224242,1.4768185945
B,0,-2.8816660357,-0.4271020305,-0.1397756431
F,0,-2.4604990959,0.1120606389,1.0798961407
F,0,-2.3365674159,0.3725174101,-1.1962687035
F,0,-4.2766948179,-0.3767614575,-0.2332136412
F,0,-2.4349502051,-1.7397302138,-0.2825746772
H,0,2.6228425881,2.2662900274,1.1304504511
----

8 (R^1 = Me, R^2 = Me)

P,0,2.2696052661,-0.2186566037,-0.1273460944
O,0,-0.6239320345,-2.6549960992,0.0357803428
O,0,0.8103664866,-0.2628489047,-0.7391158161
O,0,2.3635215308,0.0558882395,1.3369344892
C,0,2.9922254487,-1.7681363629,-0.6351739844
H,0,2.4801427238,-2.582020483,-0.1181979202
H,0,2.8934225573,-1.8954697295,-1.7138106359
H,0,4.0475580382,-1.7809736136,-0.3564945802
C,0,-0.1026813948,2.5444315122,0.7727228863
H,0,0.9242503364,2.8228264029,0.5345717427
H,0,-0.1152912572,1.9399700828,1.682437339
H,0,-0.6972376683,3.4475128183,0.9222185378
O,0,-0.6092420892,1.8010514532,-0.3418225667
H,0,-1.5099496949,1.51171806,-0.1290280734
O,0,2.9600114639,0.9477546339,-1.0022951028
H,0,0.2258347584,0.5660260832,-0.5930934153
C,0,4.1910645581,1.5251458374,-0.5359138103
H,0,4.9511678151,0.7525588919,-0.395866095
H,0,4.5184367413,2.2190436676,-1.3073883967
H,0,-0.1945477415,-1.8545994345,-0.2995263896
H,0,-1.463789925,-2.3370984805,0.3918305997
B,0,-3.3039069627,-0.2957517609,0.2332673509
F,0,-3.0037028747,-1.3732909366,1.0885790317
F,0,-2.9681777105,0.9181013804,0.8812160163
F,0,-4.6605598803,-0.2999619631,-0.0832906908
F,0,-2.523372837,-0.3981220023,-0.929017262
H,0,4.0265463459,2.0605343112,0.4001444975
----

7 (R^1 = Me, R^2 = Bu)

P,0,-0.3872732269,-1.6670166348,0.3793732291
O,0,-1.6123199662,-2.3624624537,1.1579950483
O,0,-0.9203705984,-1.4101734596,-1.112272643
O,0,0.101588257,-0.4411105462,1.0728501171
C,0,0.7815204822,-2.98935549,0.1679084626
H,0,1.2320374935,-3.2190524033,1.134633061
H,0,0.2765897867,-3.8728152332,-0.2252220941
H,0,1.552777526,-2.6651983051,-0.531794409
C,0,0.5824304362,2.6231679465,-0.4030716393
H,0,1.5846475844,2.8739893454,-0.7541577595
H,0,0.6481885791,2.2150154088,0.6088704194
H,0,-0.0207826228,3.5356236352,-0.3867193416
O,0,0.0362997599,1.6690772887,-1.3100183926
H,0,-0.8259728765,1.4078872352,-0.9588023793
O,0,1.8152934924,-0.4163389318,-1.9401095254
H,0,1.1021458402,0.1887024634,-1.6700213514
C,0,3.0509670869,0.2170039,-1.6330736078
H,0,3.8293657426,-0.3444297439,-2.1583922346
H,0,3.0599535879,1.240149806,-2.0322670917
C,0,3.3426380356,0.2364629252,-0.1406948821
H,0,2.539348705,0.7745937202,0.3743899959
H,0,3.3279318852,-0.7932933407,0.2369694031
C,0,4.6865491422,0.8899555138,0.1718309243
H,0,5.4812126346,0.3620922899,-0.3676311491
H,0,4.6837265364,1.9189683126,-0.2058891207
C,0,4.9990768203,0.8944859302,1.6653568854
H,0,5.0394613315,-0.1257404047,2.0582774551
H,0,5.960645219,1.370838588,1.8729395574
H,0,4.2289326448,1.4351644188,2.2230406096
H,0,-1.739042912,-0.8818894601,-1.1612201036
H,0,-2.2509690924,-1.7117024583,1.5006032686
B,0,-3.3272591131,0.7664682355,0.4032300588
F,0,-3.0946247411,-0.0304141048,1.5450058382
F,0,-2.3043781448,1.7201440994,0.2822470495
F,0,-4.5656277015,1.3867946304,0.4959599995
F,0,-3.2930266138,-0.0746607229,-0.7383876581

TS3 (R^1 = Me, R^2 = Bu)

P,0,-0.5917288773,1.6665087177,0.12649159
O,0,0.8660012357,3.2341763149,0.9430439525
O,0,0.8581354598,1.0072710387,0.2119378945
O,0,-1.4744276206,1.7658309175,1.3309596749
C,0,-0.8086408958,2.88135186,-1.1841115195
H,0,-1.5945615665,2.5557680916,-1.8676563924
H,0,-1.0665974797,3.8421780055,-0.7443816244
H,0,0.1282304192,2.9778285889,-1.7344316852
C,0,0.1790929803,-2.1207330397,0.9183590711
H,0,-0.8146085782,-2.4699609888,1.1876543765
H,0,0.4711715572,-1.262482323,1.5211652994
H,0,0.9013163518,-2.9276035878,1.0064768893
O,0,0.116058469,-1.7344109981,-0.4809599319
H,0,1.0168268522,-1.5691153327,-0.8483365507
O,0,-1.1922171142,0.2915551836,-0.7176411817
H,0,-0.4823332357,-0.8213239602,-0.6112873894
C,0,-2.6161689175,0.1338767769,-0.9080193659
H,0,-3.0972451401,1.1028445057,-0.75096903
H,0,-2.7606428121,-0.1572888735,-1.9507872616
C,0,-3.1835402305,-0.902461876,0.0424875936
H,0,-2.6979005474,-1.8671327668,-0.1451579872
H,0,-2.9444364672,-0.6051058863,1.0687815016
C,0,-4.694538178,-1.0461852452,-0.1239175387
H,0,-5.1702841273,-0.0757516478,0.0534633637
H,0,-4.9221726125,-1.3247911739,-1.1585666595
C,0,-5.2779714382,-2.085494355,0.8284801398
H,0,-5.0855089019,-1.8106507355,1.869456482
H,0,-6.3590125275,-2.1810083729,0.7019166738

H,0,-4.8325550977,-3.069047487,0.6534012339
H,0,1.3119315702,1.8513743464,0.6245486639
H,0,0.6708008718,3.3504295008,1.8779306065
B,0,3.565344089,-1.0773219376,-0.6764649756
F,0,3.1024566464,-1.4005501582,0.6043676993
F,0,2.4728602941,-1.2579193501,-1.5868137319
F,0,4.6017002654,-1.9336498143,-1.0509381327
F,0,3.9811713039,0.2510400623,-0.7303587481
----

8 (R^1 = Me, R^2 = Bu)

P,0,-1.1238675192,1.5529644329,0.4703907188
O,0,2.381424311,2.0180632489,-0.9492299051
O,0,0.2984553351,0.9726725049,0.85933463
O,0,-1.669455559,2.4759388249,1.5051753363
C,0,-0.9960742555,2.2871308675,-1.1571491879
H,0,-1.9783050582,2.6365250302,-1.4809719455
H,0,-0.3172824914,3.1403984109,-1.1049203864
H,0,-0.6113586374,1.5536450291,-1.8684038908
C,0,0.75393003,-2.2416826155,0.2094954227
H,0,-0.3062851246,-2.3100991086,0.451828399
H,0,1.3367634169,-2.2149053897,1.133426881
H,0,1.046152673,-3.1084016652,-0.3861017194
O,0,0.9381186666,-1.0384259511,-0.5442398725
H,0,1.8758829952,-0.97812084,-0.7877435899
O,0,-1.9686199702,0.193142497,0.2933497873
H,0,0.583399256,0.1586267967,0.3079145247
C,0,-3.366410995,0.2507387109,-0.0658294513
H,0,-3.88143123,0.9329745532,0.6173047886
H,0,-3.4523908472,0.635082502,-1.0873146371
C,0,-3.9348996885,-1.1476246115,0.0339122316
H,0,-3.3832329464,-1.805207772,-0.6466209002
H,0,-3.7787505157,-1.5206572996,1.0514783549
C,0,-5.4231785553,-1.1725134088,-0.3066811597
H,0,-5.9600203993,-0.5045853542,0.3754297903
H,0,-5.5718279741,-0.7763076779,-1.316929397
C,0,-6.0099949732,-2.5775100108,-0.2154839036
H,0,-5.8914644147,-2.9856419965,0.7923772131
H,0,-7.07630513,-2.5794630715,-0.4541025996
H,0,-5.5097577584,-3.2565491661,-0.9118970351
H,0,1.8943950461,1.8094743951,-0.1410890398
H,0,3.2875888141,1.7380883144,-0.7669167664
B,0,4.2476130992,-0.4636357249,0.2844900474
F,0,3.2130331743,-0.056084514,1.1373355407
F,0,3.688297786,-1.246724655,-0.7574701067
F,0,5.1946511892,-1.213133222,0.9777515877
F,0,4.8529752502,0.6746559364,-0.2855507599
----

7 (R^1 = Et, R^2 = Bu)

P,0,0.5965178941,1.7621702377,0.4683249079
O,0,1.7081081399,2.3179329148,1.2945419872
O,0,1.0973773984,1.2701095049,-0.9792714603
O,0,-0.1302955234,0.4935470681,1.1305328571
C,0,-0.7667765649,2.8618539537,0.1184035892
H,0,-1.4285242087,2.3344428192,-0.5725707962
H,0,-1.3010517061,3.0000530209,1.062679778
H,0,0.4728042837,-0.2599975443,1.2629751629
C,0,-0.549640984,-2.7879228993,-0.1526939533
H,0,-1.590556661,-3.0586349029,-0.3375191682
H,0,-0.4638919615,-2.3742688025,0.8569245225
H,0,0.0622256939,-3.6920310594,-0.2196411736
O,0,-0.1679247393,-1.8409744101,-1.1439043401
H,0,0.7969044154,-1.7789321414,-1.1500065385
O,0,-1.7701536156,0.3274044646,-1.8888574872
H,0,-1.0412009945,-0.2413192107,-1.5888428739
C,0,-2.970351068,-0.3788374238,-1.5975274923
H,0,-3.7780820962,0.1458205748,-2.1161354701
H,0,-2.9166545574,-1.3938650919,-2.0137148217
C,0,-3.2581344294,-0.4448160981,-0.1055104939
H,0,-2.4011613455,-0.9023101579,0.4003768007
H,0,-3.3505725893,0.5743333044,0.2885271766
C,0,-4.5224652332,-1.2426765121,0.2032833621
H,0,-5.380007636,-0.7714706848,-0.2899045914
H,0,-4.428792765,-2.2462912693,-0.2272522329
C,0,-4.787661339,-1.3544134439,1.7016670198
H,0,-4.9027305401,-0.3649040549,2.1537416365
H,0,-5.6974372282,-1.9240584358,1.9074058622
H,0,-3.9574317232,-1.8565478588,2.2074882157
H,0,2.012219058,0.9345150476,-0.9616181799
C,0,-0.2954458108,4.1987688664,-0.4546194161
H,0,0.3560934595,4.7235638789,0.2468478735
H,0,0.2492637058,4.0575696258,-1.3906843188
B,0,3.0945145986,-1.3251961756,0.130906362
F,0,2.6413030809,-2.0402310991,-0.9948093715
F,0,3.4532073425,-0.0227524429,-0.2729921248
F,0,4.1781386211,-1.9662782829,0.7115266128
F,0,2.0271835929,-1.2382004139,1.0573776045
H,0,-1.1575039645,4.8357871344,-0.6591260267

TS3 (R^1 = Et, R^2 = Bu)

P,0,0.8572175041,1.6059333051,-0.6085459885
O,0,0.5986422555,1.2705859353,-2.0757756867
O,0,-0.8878119358,1.4969438201,-0.1444571822
O,0,2.599503226,1.7372302484,-0.7628646783
C,0,0.8520261845,3.2780797168,0.150693826
H,0,1.7124001943,3.832822628,-0.2206557718
H,0,-0.0553935771,3.7815876281,-0.1827370938
H,0,2.9161173049,1.1308935339,-1.4406298161
C,0,-0.6938247394,-1.3714384524,2.432673143
H,0,0.0723075991,-1.1390491234,3.1667685081
H,0,-0.3007260205,-1.9531532668,1.6020414215
H,0,-1.5362281785,-1.8731950553,2.9018823495
O,0,-1.1898623601,-0.0948627572,1.9335934021
H,0,-2.0758233472,-0.1854639076,1.4969820513
O,0,1.0025342432,0.3748074682,0.5314132435
H,0,-0.57065669,0.3124751791,1.2421834655
C,0,2.1555007481,-0.435301401,0.7916170196

H,0,3.0461669312,0.1887552945,0.8609567665
H,0,1.9755413254,-0.8808171017,1.7737118687
C,0,2.3202462915,-1.5233216866,-0.25427968
H,0,1.3746749908,-2.0700594352,-0.3470092386
H,0,2.5191655176,-1.0691900656,-1.2315346641
C,0,3.4496449822,-2.4859941285,0.1051920097
H,0,4.3744081465,-1.9189933909,0.2605956265
H,0,3.2180190593,-2.9733859467,1.0588612101
C,0,3.6743579034,-3.5449141891,-0.9698367799
H,0,3.953443454,-3.0829807621,-1.9210990619
H,0,4.4716149338,-4.2367150336,-0.686679379
H,0,2.7657673343,-4.1304042554,-1.138083782
H,0,-1.3495410626,1.0172315688,-0.8428608078
C,0,0.8872575089,3.2102061916,1.6793401159
H,0,0.0240988256,2.6689632687,2.0742513736
H,0,1.7941663836,2.7111748406,2.0315629813
B,0,-3.0348780198,-1.1297661754,-0.6203483077
F,0,-3.4012524665,-0.4609763515,0.5978019429
F,0,-2.8592482324,-0.1646313736,-1.6157928781
F,0,-4.0445919043,-2.0200537607,-0.9634646999
F,0,-1.8313277592,-1.8015860282,-0.3790499332
H,0,0.8738224456,4.2176780213,2.1025221044
----

8 (R^1 = Et, R^2 = Bu)

P,0,-2.7831139749,1.7019783095,0.1008799922
O,0,-3.3894495283,1.2251135538,1.3764067258
O,0,0.6209248904,0.7471088611,1.1908369193
O,0,-3.9116114349,2.1169830257,-0.9810713893
C,0,-1.6529635312,3.0808647079,0.2405709107
H,0,-2.243455191,3.9009054606,0.6609888282
H,0,-0.9098948521,2.7972007875,0.9901725163
H,0,-4.7280633371,2.4500365482,-0.5833081487
C,0,0.655389326,-0.1119815957,-2.3833860899
H,0,-0.2364059282,0.4167911356,-2.7197685729
H,0,0.3556337852,-1.0136266871,-1.8400886229
H,0,1.2460156452,-0.4013604866,-3.2575814852
O,0,1.3902855064,0.776274426,-1.5460524737
H,0,2.2151098523,0.3274382002,-1.31370836
O,0,-1.9493578168,0.6212316001,-0.7197760879
H,0,0.7315969555,0.9117222596,0.2385816386
C,0,-2.4542577555,-0.7258586467,-0.8955787052
H,0,-3.5476778216,-0.7191530261,-0.8443720159
H,0,-2.1619127314,-1.0169450812,-1.9060943581
C,0,-1.8574296684,-1.6559653953,0.1401382204
H,0,-0.766461047,-1.5728187436,0.1036794444
H,0,-2.175361535,-1.3359563462,1.1381932402
C,0,-2.2742279831,-3.1040590909,-0.1058488037
H,0,-3.3674446014,-3.1766501631,-0.1018083874
H,0,-1.9415582241,-3.411522413,-1.1035244469
C,0,-1.6924720442,-4.0484779101,0.9418837952
H,0,-2.0339241669,-3.7750159185,1.9442966639
H,0,-1.9895605417,-5.0837185123,0.7568205421
H,0,-0.5995133558,-4.0065234355,0.9387552408
H,0,0.8823552827,-0.1787345196,1.2775642711
C,0,-0.996205472,3.4841505652,-1.078717141
H,0,-0.3566987669,2.68583592,-1.4598837183
H,0,-1.7434411911,3.7238556137,-1.8386010314
B,0,3.0520316073,-1.7461177853,0.2856174284
F,0,3.2810829161,-1.2269642285,-1.0107216917
F,0,3.5900762007,-0.8696287905,1.2345361746
F,0,3.6453425464,-3.006255747,0.3937986308
F,0,1.6598462314,-1.856146531,0.4873684987
H,0,-0.3760272448,4.3689510789,-0.9242461516
